# Supplementary material for: What should pulmonary rehabilitation look like for people living with post-tuberculosis lung disease in the Bishkek and Chui region of the Kyrgyz Republic? A qualitative exploration
Source: BMJ Open. 2022 Feb 4;12(2):e053085. doi: 10.1136/bmjopen-2021-053085 (PMC8819799; doi:10.1136/bmjopen-2021-053085)
Supplement: Supplementary data [file bmjopen-2021-053085supp001.pdf]

Work Package 1 Patient Focus Group Schedule

The impact has PTBLD has had on their life

- When were you first diagnosed? What was it like to hear that?
- How did you expect PTBLD was going to affect you? Is there anything you wish you had been told about PTBLD? If so, what? Why? How would this have helped you?
- Did you know anyone else who had PTBLD? How are they treated by other people?
- How do you think other people have reacted to you having PTBLD? Did you tell family/friend/work colleagues? Immediately or not?
- What has most surprised you about having PTBLD? What are the differences between what people generally think about PTBLD and the actual experience of having it?
- Consider the impact on your work (employment), your domestic tasks, and leisure choices. What has been the most significant? What? It what ways? Give examples.
- What changes, if any, have you had to make to your life because of PTBLD? Prompt: employment, domestic duties, leisure activities, personal relationships?
- How would you advise others living with PTBLD?
- How do you feel about the future?

Everyday activities:

- When, in your everyday life are you most active?
- Do you prefer individual or social activities when you are being active? Why?
- Are there any activities that you would like to do? Or any that you no longer do that you would like to be able to do again?
- Other participants have talked about activities such as ball sports (e.g., basketball, volleyball, football), wushu, dancing, singing and music – what do you think about doing these activities?

Interviewer: Provide a brief overview of PR: "PR consists of an exercise and education programme. Treatment is tailored to each person and it aims to help you control the symptoms of your condition, educate you about your condition, improve general health and level of fitness, improve your ability to carry out daily activities that are important to you and improve your confidence and quality of life."

- What are your initial thoughts?
- How would you hope PR might help you? What would you hope to achieve from taking part?
- What do we need to think about in making you more comfortable to attend PR?
- Would you be sacrificing anything to be part of this? Time? Money? Energy that will stop you doing other things? What are the negative sides of doing PR?
- What do you think should be included in PR? Why? (PROMPT: psychological support, education, information, types of exercises). **Note: ask them for specific examples**
- Are there any activities or hobbies that could be useful in PR? (PROMPT: ball sports such as basketball, volleyball, football; wushu; dancing; singing and music
- What do you think the challenges might be with these activities as part of PR?
