# Supplementary material for: What should pulmonary rehabilitation look like for people living with post-tuberculosis lung disease in the Bishkek and Chui region of the Kyrgyz Republic? A qualitative exploration
Source: BMJ Open. 2022 Feb 4;12(2):e053085. doi: 10.1136/bmjopen-2021-053085 (PMC8819799; doi:10.1136/bmjopen-2021-053085)
Supplement: Supplementary data [file bmjopen-2021-053085supp002.pdf]

Work Package 2 Referrer Interview Schedule

Aim 1) General opinions about pulmonary rehabilitation

- What does the term pulmonary rehabilitation mean to you?
- What is your opinion about pulmonary rehabilitation?

  [Probing question: How important do you think it is to provide pulmonary rehabilitation?]
- Do you have any experience in rehabilitation?

  If so: What did the rehabilitation consist of?

  How long was the average course of rehabilitation?

  Roughly what percentage of patients would take part in the rehabilitation?
- What would you think about being involved in referring patients to pulmonary rehabilitation?
- What do you think about using your clinical time for pulmonary rehabilitation?
- What do you think the problems will be to referring patients to pulmonary rehabilitation?
- How do you think these problems can be resolved?

  [Probing question: What will be your reasons for referring patients to pulmonary rehabilitation?]
- How would you describe pulmonary rehabilitation to your patients?
- What characteristics will you look for when deciding whether or not to refer a patient?

  [Probing question: Are there certain types of patients you wouldn't even discuss pulmonary rehabilitation with? Why?]
- How do you know if someone is willing or ready to take part in pulmonary rehabilitation?
- Who do you think should be involved in assessing which pulmonary rehabilitation activities would help a specific patient? And why?
- What staff should be involved in providing pulmonary rehabilitation?

Aim 2) Patients' opinion of pulmonary rehabilitation

- How do you think patients will respond to your referral for pulmonary rehabilitation? Why?
- How do you think patients will get on with pulmonary rehabilitation? Why?
- What can we do to help patients complete their course of pulmonary rehabilitation?

Aim 3) Changes needed for pulmonary rehabilitation to work well in Kyrgyzstan

- What physical activities/exercise are good for these patients? Why? How might we do these activities as part of pulmonary rehabilitation?
- What do you think should be included in pulmonary rehabilitation? Why?

  [PROMPT: Education, information, types of exercises (e.g., walking, ball sports, cycling, yoga), psychological assistance, music]

  [Probing question: Why? Why not other activities?]

- What problems are caused by the stigma associated with PTBLD?

  [Probing question: How might these problems impact participation in pulmonary rehabilitation? What can we do to help patients take part? How can we help them to have a positive experience?]

  [PROMPT: environment, social influence, emotional conditions, feeling comfortable]

- Should pulmonary rehabilitation address problems about stigma?

  [Probing question: How might pulmonary rehabilitation reduce stigma?]
