# Supplementary figures and images for: What should pulmonary rehabilitation look like for people living with post-tuberculosis lung disease in the Bishkek and Chui region of the Kyrgyz Republic? A qualitative exploration
Source: BMJ Open. 2022 Feb 4;12(2):e053085. doi: 10.1136/bmjopen-2021-053085 (PMC8819799; doi:10.1136/bmjopen-2021-053085)

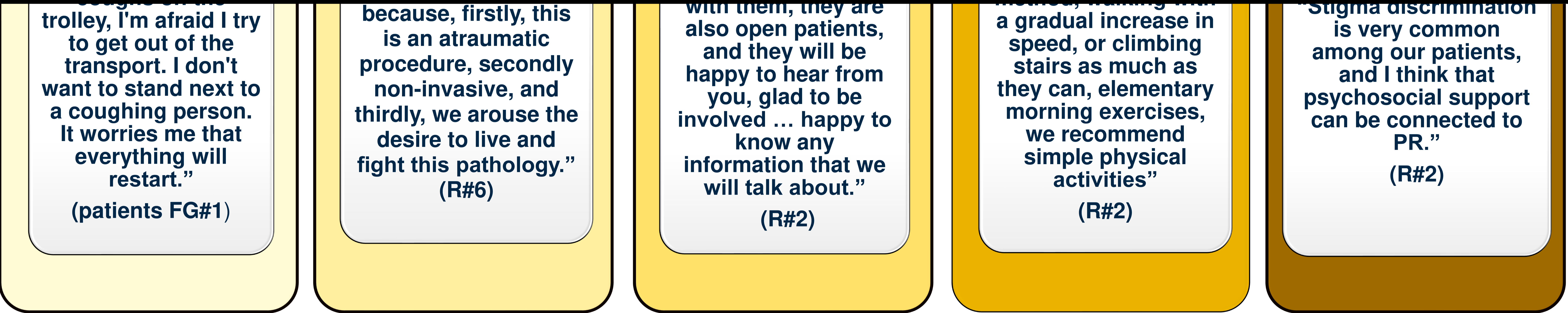

Supplement: Supplementary data [file bmjopen-2021-053085supp003.pdf]
