# Supplementary material for: What should pulmonary rehabilitation look like for people living with post-tuberculosis lung disease in the Bishkek and Chui region of the Kyrgyz Republic? A qualitative exploration
Source: BMJ Open. 2022 Feb 4;12(2):e053085. doi: 10.1136/bmjopen-2021-053085 (PMC8819799; doi:10.1136/bmjopen-2021-053085)
Supplement: Author's manuscript [file bmjopen-2021-053085.draft_revisions.pdf]

BMJ Open is committed to open peer review. As part of this commitment we make the peer review history of every article we publish publicly available.

When an article is published we post the peer reviewers' comments and the authors' responses online. We also post the versions of the paper that were used during peer review. These are the versions that the peer review comments apply to.

The versions of the paper that follow are the versions that were submitted during the peer review process. They are not the versions of record or the final published versions. They should not be cited or distributed as the published version of this manuscript.

BMJ Open is an open access journal and the full, final, typeset and author-corrected version of record of the manuscript is available on our site with no access controls, subscription charges or pay-per-view fees (http://bmjopen.bmj.com).

If you have any questions on BMJ Open's open peer review process please email info.bmjopen@bmj.com

BMJ Open

What should pulmonary rehabilitation look like for people living with post-tuberculosis lung disease in Kyrgyzstan?: A qualitative exploration

Journal:	*BMJ Open*
Manuscript ID	bmjopen-2021-053085
Article Type:	Original research
Date Submitted by the Author:	22-May-2021
Complete List of Authors:	Mademilov, Maamed; National Center for Cardiology and Therapy named after academician Mirsaid Mirrakhimov under the Ministry of Health of the Kyrgyz Republic, Respiratory medicine<br>Mirzalieva, Gulzada; National Center for Cardiology and Therapy named after academician Mirsaid Mirrakhimov under the Ministry of Health of the Kyrgyz Republic, Respiratory medicine<br>Orme, Mark; University of Leicester, Department of Respiratory Sciences; University Hospitals of Leicester NHS Trust, Centre for Exercise and Rehabilitation Science, NIHR Leicester Biomedical Research Centre – Respiratory<br>Bourne, Claire; University Hospitals of Leicester NHS Trust, Centre for Exercise and Rehabilitation Science, NIHR Leicester Biomedical Research Centre – Respiratory<br>Akylbekov, Azamat; National Center for Cardiology and Therapy named after academician Mirsaid Mirrakhimov under the Ministry of Health of the Kyrgyz Republic, Respiratory medicine<br>Jones, Amy; University of Leicester, Department of Respiratory Sciences; University Hospitals of Leicester NHS Trust, Centre for Exercise and Rehabilitation Science, NIHR Leicester Biomedical Research Centre – Respiratory<br>Miah, Ruhme ; University of Leicester, Department of Respiratory Sciences; University Hospitals of Leicester NHS Trust, Centre for Exercise and Rehabilitation Science, NIHR Leicester Biomedical Research Centre – Respiratory<br>Jones, Rupert; University of Plymouth, Faculty of Health<br>Barton, Andy; University of Leicester, Department of Respiratory Sciences<br>Malcolm, Dominic; Loughborough University, School of Sport, Exercise and Health Sciences<br>Sooronbaev, Talant; National Center for Cardiology and Therapy named after academician Mirsaid Mirrakhimov under the Ministry of Health of the Kyrgyz Republic, Respiratory medicine<br>Singh, Sally; University of Leicester, Department of Respiratory Sciences; University Hospitals of Leicester NHS Trust, Centre for Exercise and Rehabilitation Science, NIHR Leicester Biomedical Research Centre – Respiratory<br>Yusuf, Zainab; University of Leicester Department of Respiratory Sciences, Department of Respiratory Sciences ; University Hospitals of Leicester NHS Trust, Centre for Exercise and Rehabilitation Science,

NIHR Leicester Biomedical Research Centre – Respiratory, University Hospitals of Leicester NHS Trust

Keywords: Tuberculosis < INFECTIOUS DISEASES, Respiratory infections < THORACIC MEDICINE, REHABILITATION MEDICINE, RESPIRATORY MEDICINE (see Thoracic Medicine), QUALITATIVE RESEARCH

SCHOLARONE™ Manuscripts

I, the Submitting Author has the right to grant and does grant on behalf of all authors of the Work (as defined in the below author licence), an exclusive licence and/or a non-exclusive licence for contributions from authors who are: i) UK Crown employees; ii) where BMJ has agreed a CC-BY licence shall apply, and/or iii) in accordance with the terms applicable for US Federal Government officers or employees acting as part of their official duties; on a worldwide, perpetual, irrevocable, royalty-free basis to BMJ Publishing Group Ltd ("BMJ") its licensees and where the relevant Journal is co-owned by BMJ to the co-owners of the Journal, to publish the Work in this journal and any other BMJ products and to exploit all rights, as set out in our licence.

The Submitting Author accepts and understands that any supply made under these terms is made by BMJ to the Submitting Author unless you are acting as an employee on behalf of your employer or a postgraduate student of an affiliated institution which is paying any applicable article publishing charge ("APC") for Open Access articles. Where the Submitting Author wishes to make the Work available on an Open Access basis (and intends to pay the relevant APC), the terms of reuse of such Open Access shall be governed by a Creative Commons licence – details of these licences and which Creative Commons licence will apply to this Work are set out in our licence referred to above.

Other than as permitted in any relevant BMJ Author's Self Archiving Policies, I confirm this Work has not been accepted for publication elsewhere, is not being considered for publication elsewhere and does not duplicate material already published. I confirm all authors consent to publication of this Work and authorise the granting of this licence.

For peer review only

What should pulmonary rehabilitation look like for people living with post-tuberculosis lung disease in Kyrgyzstan?: A qualitative exploration

Author affiliations

M. Mademilov[1]*, G. Mirzalieva[1]*, M.W. Orme[2,3], C.L.A. Bourne[3], A. Akylbekov[1], A.V. Jones[2,3], R.B. Miah[2,3], R. Jones[4], A. Barton[2], D. Malcolm[5], T. Sooronbaev[1], S.J. Singh[2,3], Z.K. Yusuf[2,3]

*Authors contributed equally.

Correspondence to Dr. Maamed Mademilov; mademilov@gmail.com

[1] National Centre of Cardiology and Internal Medicine (NCCIM) named after academician M. Mirrakhimov - Bishkek (Kyrgyzstan)

[2] Department of Respiratory Sciences, University of Leicester (United Kingdom)

[3] Centre for Exercise and Rehabilitation Science, NIHR Leicester Biomedical Research Centre – Respiratory, University Hospitals of Leicester NHS Trust - Leicester (United Kingdom)

[4] Faculty of Health, University of Plymouth (United Kingdom)

[5] School of Sport, Exercise and Health Sciences, Loughborough University (United Kingdom)

Abstract

Objective

After experiencing TB, many people develop post-tuberculosis lung disease (PTBLD). Pulmonary rehabilitation (PR) centrally comprising of education and exercise is recommended internationally for people living with chronic respiratory diseases (CRDs). However, no such service exists in Kyrgyzstan. This study investigated the opinions of healthcare professionals (HCPs) who would be expected to be potential future referrers to PR and adults living with PTBLD about what a PR program could look like in Kyrgyzstan.

Design

A qualitative study using interviews and focus groups. Grounded theory and thematic analysis were used for data collection and analysis.

Participants

63 participants; 15 referrers and 48 adults living with PTBLD.

Setting

Participants were recruited from hospital settings in Bishkek and Chuy Region, Kygryzstan.

Methods

Fifteen semi-structured interviews were conducted with referrers and 9 focus group discussions were conducted with adults living with PTBLD.

Results

Five key themes were developed; (i) living with PTBLD; (ii) attitude to PR, which emphasized the perceived importance and potential benefits of implemention; (iii) barriers/facilitators to PR, which included time and cost, and the importance of appropriate communication in enabling participation; (iv) interventional components of PR, which described culturally and demographically appropriate physical activities including rhythmic movements, dance, and volleyball; and (v) psychosocial support, which demonstrated the importance of psychological support for patients coping with the effects of stigma.

Conclusions

Potential referrers and adults living with PTBLD expressed their support for the implementation of PR. The culture-specific and population-specific issues highlighted in this work demonstrate the need to adapt services to the local context, potentially expanding PR components beyond international guidelines to make services attractive to those they seek to help and those involved in the delivery. The principles of culturally adapting PR may be helpful for those looking to establish similar clinical services in other LMIC and in Central Asia in particular.

Data availability statement

Data relevant to the study has been included. Further data is available upon reasonable request.

For peer review only

Strengths and limitations of this study

- In LMIC, the primary focus of CRD management is pharmacological treatment, with PR being poorly developed and not widely accessible or recognised.
- It is important to design a PR service informed by both the people it is trying to help and those involved in its future delivery, to ensure its appropriateness and to make it attractive to patients, healthcare workers and policy makers.
- Kyrgyzstan is leading the development of PR among many other post-Soviet countries and in Central Asia, where there is currently an absence of rehabilitation services, and therefore ideas about how to successfully implement PR may have significant implications for other LMIC or low-resource contexts.
- The study was conducted in one geographical region in Kyrgyzstan, therefore the implications reported need to be tested to show applicability to other cultural contexts.

For peer review only

Introduction

Worldwide, 10 million people suffer with tuberculosis (TB) and 1.5 million people die from TB annually. The burden of TB on individuals and society falls predominantly in Low- and Middle-Income Countries (LMIC)[1] where weak economic conditions aggravate the situation of people living with the disease, leading to disability and early mortality[2,3]. Kyrgyzstan is a LMIC in Central Asia with a high poverty level (20.1%)[4,5]. According to the World Health Organisation (WHO), the relative incidence rate of TB in Kyrgyzstan in 2019 was 144 per 100,000, the highest rate in the WHO European Region[6], as well as having one of the highest respiratory mortality rates[7].

Many adults who have experienced TB go on to develop post-tuberculosis lung disease (PTBLD). People with PTBLD experience decreased exercise tolerance and increased cough, shortness of breath and general weakness. This is often accompanied by feelings of social isolation and depression[8,9]. People living with PTBLD can also experience stigma due to negative social perceptions of TB and, in particular, the risk of infection to others. There is evidence of persistent stigmatization of people living with TB even after recovery[10,11]; compounding problems such as social exclusion, unemployment, unstable housing and limited access to medical care[10,12,13]. Depression, anxiety and somatic symptom disorders

have a particularly negative impact on the standard of living of people in LMIC such as Kyrgyzstan[3,14,15].

Kyrgyzstan declared its independence from the Union of Soviet Socialist Republics (USSR) when it collapsed in 1991. The Soviet model of health care, which was centralized and provided universal free access to medical care, was designed for the prevention and early warning of diseases. During the Soviet era, people living with PTBLD across the USSR attended pulmonary rehabilitation (PR), where services ranged from three weeks to three months and included breathing exercises, prescribed walking, massage and various ball games. In addition, there were other alternative treatment institutions such as sanatoriums, salt mines and alpine resorts.

After the collapse of the Soviet Union, health care systems in independent states have experienced an economic crisis, an outflow of specialists, a decrease in the quality of medical care, a shortage of drugs, and an increase in the number of diseases such as TB and Human Immunodeficiency Virus (HIV). While the rehabilitation centers opened during the Soviet era continued to operate after the independence of Kyrgyzstan, due to a lack of funding, the quality and usefulness of the services provided has reduced[16]. Rehabilitation is rarely available to patients. Consequently, the burden of TB and PTBLD has continued to grow since Soviet times[17].

The importance of PR is well evidenced for people living with chronic respiratory diseases (CRDs), including for adults living with PTBLD[10,18,19]. PR involves initial assessment followed by individualized therapy (exercise training and education) to improve physical and psychological well-being, and develop a long-term commitment to healthy behavior[20,21]. PR improves shortness of breath, exercise tolerance, and quality of life and reduces the number and duration of hospitalizations linked to exacerbations[18,22,23].

However, the introduction of PR in LMIC needs to be sensitive to both the local populations receiving care and the local context. Access to PR is typically through a referral process whereby healthcare professionals (HCPs) introduce PR to patients. In many LMIC, including Kyrgyzstan, Pulmonologists and TB Specialists work directly with adults living with PTBLD and will likely be involved in referring patients for PR. Consequently, this study combines the

For peer review only

views of potential PR referrers and adults living with PTBLD in Kyrgyzstan to identify both the potential demand and most appropriate design for PR.

Aims

To explore the views and experiences of potential referrers and adults living with PTBLD in Kyrgyzstan to inform the development of a culturally-appropriate PR service.

Methods

Patient and Public Involvement

As respiratory clinicians, we drew on our experiences of talking to people living with PTBLD when designing the study, in particular the interview schedule and logistics. Interviews and focus groups were organized based on work schedules and family commitments. This work was conducted to provide more in-depth insight into the views of patients to improve the quality of care they receive, such as the provision of appropriate PTBLD-specific PR. The findings of this study will be disseminated to participants and the wider public through awareness-raising activities in TB hospitals to help boost the profile of PR and inform the wider public about PTBLD-specific rehabilitation. The findings will also be disseminated through national and international conferences. The results of this study are expected to inform the Kyrgyz healthcare system by introducing a culturally-appropriate rehabilitation service informed by patients themselves.

Participants and data collection

HCPs in regular contact with adults living with PTBLD were invited to participate and were recruited from 5 sites (National Hospital, National TB Hospital, City TB Hospital, Chuy region TB Hospital & Alamedin District TB Hospital). A total of 17 HCPs were invited to take part. Two HCPs declined to participate due to lack of time, leaving 15 individuals who participated in semi-structured interviews (12 females, 3 males; 3 Pulmonologists and 12 TB Specialists).

Participants for focus group (FG) discussions were invited from 8 sites (National Hospital, National TB Hospital, NCCIM, City TB Hospital, Chuy region TB Hospital, Sokuluk District TB Hospital, Alamedin District TB Hospital & Kant District TB Hospital). In total, 9 FGs were organized. 80 people were invited to participate, of whom 48 took part (26 men and 22 women) in groups of between 4 and 8.

For peer review only

All participants provided written informed consent. The interviews with referrers were conducted face-to-face at the participants' workplace between October 2019 and March 2020. The FGs were conducted on the premises of the department of respiratory medicine of the NCCIM between October 2019 and January 2020.

The interviewer (MM) was a Pulmonologist in Bishkek, Kyrgyzstan with previous experience in qualitative research. MM did not have any relationship with participants prior to study commencement and equally, participants did not know MM prior to the interviews.

The interview guides were structured using a narrative approach with additional prompts. To improve and adapt questions and prompts, notes were taken and discussions of transcribed data were regularly held with the research team. The interviews and FGs were conducted in Russian and Kyrgyz languages according to participant preference. The researchers used audio recordings to collect the data. The average interview duration was 30 minutes, and for FGs 50 minutes. There were no follow-up interviews or FGs.

The Kyrgyz research team transcribed the recordings of the interviews verbatim and then translated the transcripts into English for the purpose of triangulation with wider study team members. Each interview and FG transcript was assigned a sequence number. Transcripts were anonymized and were not returned to participants for verification.

Data Analysis

The theoretical framework used was grounded theory and the data were analyzed using thematic analysis[24]. The flexibility and explorative character of thematic analysis makes it particularly appropriate for comparing and contrasting qualitative data drawn from different sample groups, and where researchers have variable levels of understanding of the life-worlds of different participant groups.

Each step of the analysis was carried out independently by the Kyrgyz and UK teams, followed by joint discussions, and agreement on the key themes. The first step involved reading and re-reading the transcripts several times (in their original or translated form), and writing a brief summary of the data. Step two involved creating codes. There were seven coders in total; three in Kyrgyzstan, and four in the UK. The combination of similar phrases and sentences were highlighted in the same color, placed under the same code and a description of its contents was developed. A total of 21 initial codes were created. The

For peer review only

third step involved refining the themes by grouping together similar codes. The fourth step involved collating all of the relevant quotes and clarifying how each data extract related to the different themes. The fifth step involved developing a detailed description of themes and subthemes together with the use of relevant quotes.

Summary findings and interpretations encapsulating the perspectives of both referrers and adults living with PTBLD are presented. For the purposes of this article, data have been organized into five overarching themes, impacting a range of different PR-related issues. Quotes were selected from frequently encountered codes during the interviews and FGs and chosen as the best representation of the analytic point.

Results

Referrers and respondents living with PTBLD were positive about the potential introduction of PR. They identified a range of barriers/facilitators that would need to be considered in the design and implementation of any such program. A range of potential PR components were identified, as well as the need to tailor PR to individual physical conditions, provide effective communication and education, and be sensitive to the patients' psychosocial state. Respondents living with PTBLD shared their illness experiences, such as symptom burden, challenges faced, and patterns of exercise and physical activity, and provided a vision of how a PR program should best be designed.

Living with PTBLD

Respondents living with PTBLD identified a range of expected symptoms including, "weakness and shortness of breath" (#FG2). However, the severity of symptoms varies for people living with PTBLD and affects their daily life in different ways. For some, symptoms do not present any difficulties for day-to-day living; "At the moment I am walking freely, doing household chores" (#FG5). But how strongly the respiratory symptoms of PTBLD affect quality of life was noted by another respondent: "...with any physical movement I have shortness of breath... shortness of breath is strong" (#FG9).

Many respondents living with PTBLD worry about contracting TB again. They strictly monitor their symptoms and when their condition worsens or new symptoms emerge, they turn to medical staff for advice:

For peer review only

> "…I do everything that the doctor said, every year I go to the clinic to [the] TB specialist, cough, sneeze, get a yearly consultation, every year I go to X-rays" (#FG1)

In addition to recognizing the efficacy of traditional treatments, some respondents resort to alternative treatments; "…I drink rose hips, sometimes with lemon, I eat a spoonful of honey all the time, I try to drink milk, yogurt" (#FG1). Many respondents choose spa treatment: "… I went to the salt room, in the sanatorium, Kyrgyz seaside" (#FG1). Others noted that movement and physical exercise in general improved their well-being:

> "I…try to walk, sometimes I walk from morning to evening. I believe that walking, in general, physical activity is a huge benefit" (#FG5)

Particularly in rural communities, where agricultural work is common, day-to-day living is relatively active. Others noted how activity was embedded into their lifestyle - "I walk a lot, do not use the washing machine, cook and wash" (#FG5) – or did certain activities at home: "…I do breathing exercises so as not to cough. In these moments, gymnastics helps me" (#FG2).

Respondents have become accustomed to living with respiratory symptoms; "I wanted to live and breathe deeply, we have already forgotten what it is, but we want it so much" (#FG1). They are frequently proactive, seeking to improve their condition; "…we make different attempts, we want to get better. We need life" (#FG2). Consequently there is a significant demand for the implementation of a service like PR, which can help improve health-related quality of living.

Attitude to PR

All referrers noted the importance of implementing PR and recognized that the current absence of services meant that, post-treatment, people living with PTBLD were left without effective guidance:

> "Rehabilitation is the process necessary after completion of treatment for complete recovery" (#R12)

This sense of importance was confirmed by the FG data, whereby participants noted the potential of PR for improving their quality of life, increasing exercise tolerance and reducing the severity of symptoms: "If I constantly move, I feel good, nothing hurts" (#FG8).

For peer review only

However, some have a limited awareness about which types of activities are suitable - “…you can or cannot play sports…, I don’t know…" (#FG4) - or how to participate in these activities safely; "…we don't know what movements to do and how to do them correctly" (#FG2).

Consequently, FG respondents noted the importance of receiving trustworthy, expert advice about PR and qualified assistance from medical personnel; “health professionals need to be trained so that I can come and ask, and not receive the answers that I know" (#FG1). HCPs should also adapt PR to individual needs: "The main thing is that the coach is good, that he knows how to give the right load" (#FG5).

Referrers noted the value of helping people to improve physical function and adapt to social life. As one referrer noted, PR would:

> “[lead to] an increase in tolerance to physical exertion, it would be like an addition to the main treatment… then it would reduce the number of complications, the number of exacerbations in general... so that the diseases do not become more complicated, do not worsen, as it seems to me that PR has a huge role [to play].” (#R9)

Respondents living with PTBLD generally exhibited a positive attitude towards PR with many expressing an interest in participating: "I will gladly come" (#FG1). Another respondent stated: “Yes, absolutely necessary" (#FG3). The FGs also revealed that some people might not want to take part in PR; for example, people who believe they are relatively healthy or have mild symptoms may be uninterested and see no benefit in participating.

Barriers/Facilitators to PR

HCPs identified relatively few barriers to referring patients to PR. Only one fundamental issue was identified as precluding PR participation; namely the presence of concomitant diseases (#R11) or unstable conditions (#R9).

Referrers did however note that some personality types – “phlegmatic people” (#R8), those whose behavior was erratic, “forgetful, shy” (#R11) - were not well suited to PR and that this would affect uptake. The importance of personality type was also noted by FG respondents:

> "Among the patients there are aggressive patients who run into others for no reason, and there are calm ones on the contrary, who prefer more privacy" (#FG7)

For peer review only

A further consideration was the social situation of some adults. It was noted that many migrate after finishing treatment. It was also noted that, “among the patients with TB, we have former convicts and drug addicts" (#R10), “alcoholics, homeless people” (#R4) and so PR opportunities would not be taken up by everyone.

More generally, cost and time were identified as major considerations. A number of referrers argued that PR must be free to encourage attendance and meet the expectations of those living with PTBLD (#R5, 11, 13). FG respondents felt the same (#FG1) and emphasized the potential financial burden of implementing PR: “I wish there was a discount. Many patients do not work, there is little money…"(#FG2).

Personality types and the social situation were identified as interdependent factors. One referrer noted that attitudes from those with PTBLD were affected by the experience of having TB; “…because our patients have been treated for a long time, they are asocial for a very long time” (#R1). Others felt that the prolonged treatment period often experienced would make people reluctant to volunteer for further engagement. Some referrers noted that adherence could be affected by slow positive effects on health, which can lead to a loss of interest:

> “…they don’t feel [the effects] right away… gradually if this is repeated regularly, you will feel a positive effect” (#R2)

The two primary solutions proposed for these issues were educating the patient with a “conversation about the need for post-treatment rehabilitation” (#R1), and attending to the “dynamics” and “subjective feelings” (#R2) generated in the PR environment that will help strengthen adherence to PR.

Referrers talked about the need for effective doctor-patient communication for the success of PR. In the context of referring a patient to PR, interviewees noted that the authority and recommendations of the attending physician will have a very important impact on a person’s decision to participate in PR:

> “If you explain normally to a person, any person will do what the doctor recommends, because in our society, as a doctor, as long as we have authority, patients believe us.” (#R3)

For peer review only

Most respondents noted that they would follow recommendations to improve their health:

> "You need to do whatever your doctors tell you, take the right medication, eat right" (#FG3).

However, positive clinician-patient relations cannot be taken for granted as some of the FG respondents recalled unfavourable experiences with medical staff in the past. Being open and friendly was seen as an important source of motivation for those living with PTBLD;

> "…if you communicate openly with them, they are also open patients, and they will be happy to hear from you… happy to know any information." (#R2)

However, this alone would not be sufficient to mitigate experiences of stigma, which created a closed-minded attitude amongst those affected. Referrers explained how this could affect interactions with other HCPs:

> "…because even if we write a certificate for them, … [that] they are not contagious to others, sometimes in the consultation department or even in the hospital they look at patients differently and say 'you are ill with TB'" (#R12)

Interventional components of PR

While all referrers and FG respondents noted the importance of implementing physical exercises, a broad range of specific exercises were described. Referrers noted that walking was "the simplest and cheapest method" (#R2), while swimming and Nordic walking were commended for activating a wide range of muscles (#R9, #R15). Other activities included rhythmic movements (dancing, wushu [Chinese Kungfu]), ball sports (volleyball, basketball) and recreational activities (running, cycling, gymnastics). Like many countries, Kyrgyz culture is heavily influenced by dance, and the national dance "Kara Jorgo" ("Black Stallion") was cited as an activity that may be suitable to include in PR (#R12). Referrers argued for "…almost all the exercises that involve the respiratory muscles, [and] the muscles of the shoulder girdle ... especially the muscles of the arms and shoulders" (#R9). They further noted the "…need to try everything…" (#R5) because different people like different forms of exercise.

For peer review only

Referrers and FG respondents also supported singing as part of the rehabilitation, though there were several opposing opinions. For instance, FG participants argued that “singing songs can be added to rehabilitation… it's good for the lungs” (#FG5), while referrers noted that attention should be paid to contraindications and severe concomitant diseases, which can lead to serious consequences such as internal bleeding:

> “If they have changes in the lungs, if they have caverns, then… singing can somehow affect the deterioration of their condition” (#R1)

But of greater concern to the referrers and FG respondents was that the intensity of activity should be suitable to the individual. Exercises needed to be done, “…not at such a pace, but … sparing, light” (#R4). As one FG respondent remarked, “…don't overwork" (#FG4). Referrers rejected “…the dances where they jump intensively there, run there,” (#R9) and recommended activities such as:

> “…rhythmic dances, then slow ... they can still use the respiratory muscles in their work and [this] may, it seems to me, be useful for recovery” (#R9)

Additionally, FG respondents emphasized the importance of developing the correct schedule and suggested having both morning and evening classes because, “sometimes they [people] are not allowed to leave work" (#FG1). Others noted that the time pressures experienced by those who work or have domestic or childcare duties meant that it would be good if “some exercises can be done at home” (#R11).

Finally, referrers stated that where education was offered to those with PTBLD, verbal delivery was preferable to reading or text:

> “…video lectures should be organized in all clinical specialized pulmonary units. They should be concise, lasting 10-15 minutes” (#R6)

Despite various challenges, the strength of desire to overcome the consequences of PTBLD was evident in the respondents’ willingness to spend their limited time and money to participate in PR (#FG1). The benefits of PR were highlighted: “the time spent on recovery is not in vain. To do this, you can postpone other activities" (#FG5).

For peer review only

Psychosocial support

All TB specialists noted the need to address psychological problems (e.g. depression, becoming withdrawn or aggressive), personal and relational issues (e.g. with family) and to help people living with PTBLD adapt to social life. According to referrers, people may experience stigmatization from a variety of sources. Some people "leave the family" (#R13) due to the stigma of illness, while others are afraid that they will meet people they know at the hospital; "…[patients] tell us where and in which hospitals their relatives, friends work and do not want to visit [these places]' (#R15). Another noted that, "stigma discrimination is very common among our patients, and I think that psychosocial support can be connected to PR" (#R2).

This was supported by the FG data, where people shared their experiences of PTBLD-related stigma. Several participants said they experienced difficulties finding a job; "I tried so many times to get a job, they didn't take me" (#FG7). Some people struggle to recover and after treatment and discharge, they are subjected to stereotyping, affecting their relationships with their loved ones. For example,

> "My neighbor...told her (daughter-in-law) and the children not to come to me, that I was contagious, and to other neighbors that I needed to be evicted." (#FG3)

Indicatively, it was suggested PR should be done "anonymously so that no one knows" (#FG1).

Consequently, both referrers and FG participants placed significant emphasis on the role of psychological support within PR interventions. It was argued that some psychological support would be provided through the social nature of some PR activities. Where rehabilitation takes place in a group, all adults with PTBLD "will be equal … to each other, they will talk, they will communicate" (#R3). Conducting group PR discussions could provide mutual support because, "…until a person gets sick, they do not understand others, and there everyone understands each other, they talk, they share secrets" (#R11). There was also a call for more formal, professional psychological support; "we need psychological therapy" (#FG7).

Thus, it was not only argued that PR needed to incorporate psychological support but that it offers a good opportunity to provide the kind of support that was more generally needed by

For peer review only

people living with PTBLD. As one FG respondent noted, "The main thing is psychological support. Sport is sport, but warm words cannot be replaced by anything" (#FG5).

Discussion

The need to develop appropriate PR services for people living with PTBLD, and who reside in LMIC, is an important issue that has scarcely been studied. This qualitative study synthesized the views of adults living with PTBLD, and the TB Specialists and Pulmonologists who would initiate referral, to explore how PR could be tailored to maximize uptake in the context of Kyrgyzstan. The results indicate both a general need and willingness to embrace PR, as well as key barriers (e.g., cost, time) and facilitators (e.g. staffing, scheduling, and delivery mode) to uptake, and specific guidance on the components of intervention, including the role of psychosocial support.

At present, the treatment process for these individuals is exclusively pharmacological (lasting between 6 months and 2 years), and with no post-treatment care pathway, they are often left in a state of limbo whereby they are no longer infectious but are living with chronic symptoms and associated stigma. PR was viewed as playing an important role in improving both people's physical and mental health.

The integration of PR into the treatment pathway should be supported by strong long-term relationships between patients and clinicians, primarily a TB specialist or Pulmonologist. The data suggest that a general inclination to respect the authority of medical professionals, and a widespread compliance with treatment recommendations, will be enhanced by an open and honest attitude towards patients. Good communication by HCPs is needed to build rapport and trust with a cohort who may have experienced discrimination. There is a need to include PR in doctor-patient conversations early in the TB treatment process so that those who go on to develop PTBLD are aware that PR is a necessary and valuable part of their overall recovery and reintegration into society. Because of the contrasting medical guidance at the treatment and rehabilitation phases for TB (i.e. initially rest and latterly activity), clinicians should be transparent and consistent in promoting awareness of the benefits of PR early on during treatment. This will likely boost the uptake to PR.

In particular, continued patient engagement will be influenced by social factors. Because social attitudes prevent some patients from participating in hospital-based PR services, it is

For peer review only

vital to design a recruitment pathway and appointment logistics that are sensitive to issues relating to stigmatization. For example, it may be suitable to conduct PR classes at quieter times of the day, to help patients arrange transport or to offer overnight accommodation for those travelling far from rural regions. It would be desirable to provide advice about exercise that can be done at home and particularly about what level of exertion is both beneficial and safe. Many people living with PTBLD experience financial difficulties[25], and either struggle to find employment or secure only relatively low-paid jobs. Therefore, when organizing PR for such a population, discounted or low-cost provision is essential.

Educational components of PR should be tailored to the needs of the particular audience. In parts of the world where PR is part of standard care, education has traditionally been delivered in a lecture style but the data suggest that information provided verbally, with supporting images, and in 'bite size' amounts, would be more suitable for the PTBLD population in Kyrgyzstan. This fits with an ongoing transformation of PR education delivery in other parts of the world, such as in the UK, to move away from didactic deliveries, with the educational and cultural backgrounds of PR attendees considered[26].

Many previous studies have focused on stigma related to the infectious nature of TB and the lack of awareness within the general population that these individuals are no longer contagious after completing their drug treatment[11]. Studies conducted in LMIC such as Vietnam[27] and India[11], reported that TB is often deemed a 'death penalty' and perceived as a 'dirty disease'. Studies in LMIC have shown that the effects of stigma can both influence a patient's treatment and care-seeking behavior[28,29] and undermine patients' trust in HCPs[30]. A previous Kyrgyzstan study found that TB patients frequently experience discrimination from HCPs who sought to avoid interaction[15].

A major lesson from this study is that individuals with PTBLD may continue to experience discrimination, including from HCPs who deny them advice and make it difficult for them to visit healthcare facilities. PR offers an opportunity for these individuals to receive care from HCPs free from prejudice. Incorporating educational sessions will help them understand their disease and the social attitudes they experience. In this regard, both sets of respondents emphasized the importance of incorporating psychosocial support as part of PR to help people cope with the effects of stigma. Incorporating group sessions in a safe, non-

For peer review only

judgmental environment offers peer-peer support and will enable patients to share their experiences openly.

Another way to promote PR attendance and adherence is to embed culturally and demographically-appropriate activities. The core PR components of aerobic exercise typically include walking/cycling, strength training and education[18]. The results of this study suggest that the generic principles of PR can be adapted to the local context or population. For example, respondents spoke about the popularity of ball sports such as volleyball, reminiscent of the rehabilitation offered in the Soviet era. Conventional exercises like squats can be 'gamified' into a volleyball dig action to increase enjoyment and motivation. Adaptations are likely to be most successful where they also help patients' understanding of how these exercises can translate into benefits in daily life e.g. taking part in social activities or preparing for employment.

Respondents supported the incorporation of rhythmic movements such as dance performed at an appropriate intensity. A national dance such as Kara Jorgo, can both be scaled to each person's physical condition and offer a culturally relevant and enjoyable form of exercise to strengthen muscles involved in the mechanics of breathing. Dance has been shown to improve health, including physical performance and mood[31,32]. Taking part in socially conventional exercises will challenge stigma and show people living with PTBLD how PR is also relevant to everyday living.

In addition to dancing, singing is also an important part of the Kyrgyz culture. Unlike dancing, singing was equivocally received by research participants. Although singing therapy has been tested previously, with evidence suggesting that it can improve wellbeing for adults with COPD[33,34], evidence relating to the effects of singing for PTBLD populations is lacking. Consequently, further research is required before implementing singing as part of PR in the contexts of PTBLD and Central Asia.

Limitations

Limitations include the relatively small sample of referrers interviewed, as well as the disproportionate number of female referrers. In Kyrgyzstan, there are fewer males who specialise in TB and as a result more females were recruited. The study was conducted in one geographical region in Kyrgyzstan, therefore the implications reported may not be

For peer review only

applicable to other cultural contexts. Kyrgyzstan is leading the development of PR among many other post-Soviet countries and in Central Asia, where there is currently an absence of rehabilitation services, and therefore ideas about how to successfully implement PR may have significant implications for other LMIC or low-resource contexts.

Conclusion

PR is embedded in routine clinical care in Western countires with unequivocal evidence of its benefits. In this qualitative exploration, potential PR referrers (TB Specialists and Pulmonologists), as well as adults living with PTBLD, were overwhelmingly positive towards the implementation of PR in Kyrgyzstan. The cultural and population-specific considerations highlighted in this work demonstrate the need to adapt clinical services such as PR to the local context. The work also showcases the value of patients inputting directly into their care. Consequently, new PR services can go beyond international guidelines based almost exclusively on Western norms to ensure services are attractive to local populations whilst keeping exercise and education at its core. The act of culturally adapting PR may be beneficial for other settings looking to establish PR as a clinical service or evaluate PR in clinical trials in low resource settings.

Footnotes

Ethics statements

Ethics approval

This work is part of NIHR Global Health Research Group on Respiratory Rehabilitation (Global RECHARGE; 17/63/20), approved by The National Center for Cardiology and Internal Medicine (NCCIM; ref: 17) and University of Leicester (ref: 22293) ethics committees. This study was registered prospectively (ISRCTN16501214).

Funding: This research was funded by the National Institute for Health Research (NIHR) (17/63/20) using UK aid from the UK Government to support global health research.

For peer review only

Acknowledgments

Contributors

MM and GM undertook data collection. MM, GM, ZY and DM drafted the initial text. ZY and CB contributed to data analysis and editing of the manuscript. MO and DM contributed to the design, data analysis and editing of the manuscript. SS, MO, DM and TS contributed to the design of the study. AA, AJ, RM, RJ and AB contributed to the editing of the manuscript. All authors approved the final version to be published.

The authors express gratitude and appreciation to all of the participants involved in the study, including staff from the National Hospital, National TB Hospital, City TB Hospital, Chuy region TB Hospital, Alamedin District TB Hospital and Kant District TB Hospital.

For peer review only

References

1. Tuberculosis. *World Health Organization*, World Health Organization, 2019, www.who.int/health-topics/tuberculosis#tab=tab_1.

2. Jaramillo, E. Pulmonary tuberculosis and health-seeking behaviour: how to get a delayed diagnosis in Cali, Colombia. *Tropical medicine & international health: Trop. Med. Int. Heal.* (1998); 3.2: 138-144.

3. Peltzer, K. *et al.* Prevalence of psychological distress and associated factors in tuberculosis patients in public primary care clinics in South Africa. *BMC psychiatry* (2012);12.1: 1-9.

4. About Us: UNDP in Kyrgyz Republic. *UNDP*, 2015, www.kg.undp.org/content/kyrgyzstan/en/home/about-us.html#:~:text=Kyrgyzstan is a landlocked%2C largely,income of %241%2C170 in 2015.

5. Living standard. *National Statistical Committee of the Kyrgyz Republic*, 2019. http://www.stat.kg/en/statistics/uroven-zhizni-naseleniya/.

6. Tuberculosis Surveillance and Monitoring in Europe, 2019. *European Centre for Disease Prevention and Control*, www.ecdc.europa.eu/en/publications-data/tuberculosis-surveillance-and-monitoring-europe-2019.

7. Gibson, J., *et al.* The Burden of Lung Disease. *ERS*, 2013, www.erswhitebook.org/chapters/the-burden-of-lung-disease/.

8. Muñoz-Torrico, M. *et al.*, Is there a rationale for pulmonary rehabilitation following successful chemotherapy for tuberculosis?, *J. Bras. Pneumol.* (2016); 42.5: 374–385, 2016.

9. Cheng-Ting, L. *et al.* Evaluation of health-related quality of life in patients with tuberculosis who completed treatment in Kiribati. *Journal of International Medical Research* (2017); 45. 2: 610-620.

10. Allwood, B.W *et al.*, Post-tuberculosis lung health: Perspectives from the First International Symposium. *Int. J. Tuberc. Lung Dis.* (2020); 24. 8: 820–828.

11. Rajeswari, R. *et al.* Perceptions of tuberculosis patients about their physical, mental and social well-being: a field report from south India. *Soc. Sci. Med.* (2005); 60. 8: 1845–1853.

12. Datiko, D.G., Jerene, D., and Suarez, P. Stigma matters in ending tuberculosis: Nationwide survey of stigma in Ethiopia. *BMC Public Health* (2020); 20. 1: 1–10.

13. Bauer, M. *et al.*, Health-related quality of life and tuberculosis: A longitudinal cohort study. *Health Qual. Life Outcomes* (2015); 13.1: 1–13.

14. Walker, I. F., *et al.* Implementation of a psychosocial support package for people receiving treatment for multidrug-resistant tuberculosis in Nepal: A feasibility and acceptability study. *PloS one* (2018); 13.7: e0201163.

15. Burtscher, D. et al. 'My favourite day is sunday': Community perceptions of (drug-resistant) tuberculosis and ambulatory tuberculosis care in Kara Suu District, Osh Province, Kyrgyzstan. *PLoS One*, (2016); 11. 3: 1–16.

16. Rechel, B. Health policy reform in the countries of the former Soviet Union. *The Palgrave International Handbook of Healthcare Policy and Governance*. Palgrave Macmillan, 2015.

17. Arinaminpathy, N. and Dye, C. Health in financial crises: Economic recession and tuberculosis in Central and Eastern Europe. *J. R. Soc. Interface* (2010): 7. 52: 1559–1569, 2010.

18. McCarthy, B. *et al*. Pulmonary rehabilitation for chronic obstructive pulmonary disease. *Cochrane database Syst. Rev.* (2015); 2: CD003793.

19. Singh, S.K., *et al.* Pulmonary Rehabilitation in Patients with Chronic Lung Impairment from Pulmonary Tuberculosis. *Cureus* (2018); 10. 11.

20. Jones, R. *et al*. Does pulmonary rehabilitation alter patients' experiences of living with chronic respiratory disease? A qualitative study. *Int. J. COPD* (2018); 13: 2375–2385.

21. Corhay, J.L., *et al.* Pulmonary rehabilitation and copd: Providing patients a good environment for optimizing therapy. *Int. J. COPD* (2013); 9: 27–39.

22. Griffiths, T.L., *et al.*, Results at 1 year of outpatient multidisciplinary pulmonary rehabilitation: a randomised controlled trial. *Lancet* (2000); 355. 9201: 362–368.

23. Puhan, M.A. *et al*. Pulmonary rehabilitation following exacerbations of chronic obstructive pulmonary disease. *Cochrane Database Syst. Rev.* (2016); 12.

24. Braun, V., and Clarke, V. Using thematic analysis in psychology. *Qualitative research in psychology* (2006); 3. 2: 77-101.

25. Tanimura, T. *et al.* Financial burden for tuberculosis patients in low-and middle-income countries: a systematic review. *European Respiratory Journal* (2014); 43.6: 1763-1775.

26. Bolton, C.E., *et al.* British Thoracic Society guideline on pulmonary rehabilitation in adults: accredited by NICE. *Thorax* (2013); 68. 2: 1-30.

For peer review only

27. Hoa, N.P., *et al.* Knowledge of tuberculosis and associated health-seeking behaviour among rural Vietnamese adults with a cough for at least three weeks. *Scandinavian Journal of Public Health* 2003; 31. 62: 59-65.

28. Ngamvithayapong, J., Winkvist, A. and Diwan, V.. High AIDS awareness may cause tuberculosis patient delay: results from an HIV epidemic area, Thailand. *Aids* (2000); 14. 10: 1413-1419.

29. Weiss, M.G., Ramakrishna, J. and Somma, D. Health-related stigma: rethinking concepts and interventions. *Psychology, health & medicine* (2006); 11.3: 277-287.

30. Miller J A. Perception and beliefs of health care workers about clients with tuberculosis. Auckland: University of Auckland; 2007.

31. Koch, S.C., *et al.* Effects of dance movement therapy and dance on health-related psychological outcomes. A meta-analysis update. *Frontiers in psychology*, (2019); 10: 1806.

32. Williams, S. Dance group enriches lives of people living with breathlessness. *The Lancet Respiratory Medicine* (2020); 8. 8: 763-764.

33. Lord, V.M., *et al.* Singing classes for chronic obstructive pulmonary disease: a randomized controlled trial. *BMC Pulmonary Medicine* (2014); 14: 181-181.

34. Downes, C. *et al.* Singing for Breathing Uganda: Group singing for people with chronic lung disease in Kampala. *Journal of Applied Arts & Health* (2019); 10. 2: 219-228.

For peer review only

BMJ Open

What should pulmonary rehabilitation look like for people living with post-tuberculosis lung disease in the Bishkek and Chui region of the Kyrgyz Republic?: A qualitative exploration

Journal:	*BMJ Open*
Manuscript ID	bmjopen-2021-053085.R1
Article Type:	Original research
Date Submitted by the Author:	23-Nov-2021
Complete List of Authors:	Mademilov, Maamed; National Center for Cardiology and Therapy named after academician Mirsaid Mirrakhimov under the Ministry of Health of the Kyrgyz Republic, Respiratory medicine<br>Mirzalieva, Gulzada; National Center for Cardiology and Therapy named after academician Mirsaid Mirrakhimov under the Ministry of Health of the Kyrgyz Republic, Respiratory medicine<br>Yusuf, Zainab; University of Leicester Department of Respiratory Sciences, Department of Respiratory Sciences ; University Hospitals of Leicester NHS Trust, Centre for Exercise and Rehabilitation Science, NIHR Leicester Biomedical Research Centre – Respiratory, University Hospitals of Leicester NHS Trust<br>Orme, Mark; University of Leicester, Department of Respiratory Sciences; University Hospitals of Leicester NHS Trust, Centre for Exercise and Rehabilitation Science, NIHR Leicester Biomedical Research Centre – Respiratory<br>Bourne, Claire; University Hospitals of Leicester NHS Trust, Centre for Exercise and Rehabilitation Science, NIHR Leicester Biomedical Research Centre – Respiratory<br>Akylbekov, Azamat; National Center for Cardiology and Therapy named after academician Mirsaid Mirrakhimov under the Ministry of Health of the Kyrgyz Republic, Respiratory medicine<br>Jones, Amy; University of Leicester, Department of Respiratory Sciences; University Hospitals of Leicester NHS Trust, Centre for Exercise and Rehabilitation Science, NIHR Leicester Biomedical Research Centre – Respiratory<br>Miah, Ruhme ; University of Leicester, Department of Respiratory Sciences; University Hospitals of Leicester NHS Trust, Centre for Exercise and Rehabilitation Science, NIHR Leicester Biomedical Research Centre – Respiratory<br>Jones, Rupert; University of Plymouth, Faculty of Health<br>Barton, Andy; University of Leicester, Department of Respiratory Sciences<br>Malcolm, Dominic; Loughborough University, School of Sport, Exercise and Health Sciences<br>Sooronbaev, Talant; National Center for Cardiology and Therapy named after academician Mirsaid Mirrakhimov under the Ministry of Health of the Kyrgyz Republic, Respiratory medicine<br>Singh, Sally; University of Leicester, Department of Respiratory

	Sciences; University Hospitals of Leicester NHS Trust, Centre for Exercise and Rehabilitation Science, NIHR Leicester Biomedical Research Centre – Respiratory
**Primary Subject Heading**:	Rehabilitation medicine
Secondary Subject Heading:	Respiratory medicine
Keywords:	Tuberculosis < INFECTIOUS DISEASES, Respiratory infections < THORACIC MEDICINE, REHABILITATION MEDICINE, RESPIRATORY MEDICINE (see Thoracic Medicine), QUALITATIVE RESEARCH

SCHOLARONE™
Manuscripts

I, the Submitting Author has the right to grant and does grant on behalf of all authors of the Work (as defined in the below author licence), an exclusive licence and/or a non-exclusive licence for contributions from authors who are: i) UK Crown employees; ii) where BMJ has agreed a CC-BY licence shall apply, and/or iii) in accordance with the terms applicable for US Federal Government officers or employees acting as part of their official duties; on a worldwide, perpetual, irrevocable, royalty-free basis to BMJ Publishing Group Ltd ("BMJ") its licensees and where the relevant Journal is co-owned by BMJ to the co-owners of the Journal, to publish the Work in this journal and any other BMJ products and to exploit all rights, as set out in our licence.

The Submitting Author accepts and understands that any supply made under these terms is made by BMJ to the Submitting Author unless you are acting as an employee on behalf of your employer or a postgraduate student of an affiliated institution which is paying any applicable article publishing charge ("APC") for Open Access articles. Where the Submitting Author wishes to make the Work available on an Open Access basis (and intends to pay the relevant APC), the terms of reuse of such Open Access shall be governed by a Creative Commons licence – details of these licences and which Creative Commons licence will apply to this Work are set out in our licence referred to above.

Other than as permitted in any relevant BMJ Author's Self Archiving Policies, I confirm this Work has not been accepted for publication elsewhere, is not being considered for publication elsewhere and does not duplicate material already published. I confirm all authors consent to publication of this Work and authorise the granting of this licence.

For peer review only

What should pulmonary rehabilitation look like for people living with post-tuberculosis lung disease in the Bishkek and Chui region of the Kyrgyz Republic?: A qualitative exploration

Author affiliations

M. Mademilov[1]*, G. Mirzalieva[1]*, Z.K. Yusuf[2,3], M.W. Orme[2,3], C.L.A. Bourne[3], A. Akylbekov[1], A.V. Jones[2,3], R.B. Miah[2,3], R. Jones[4], A. Barton[2], D. Malcolm[5], T. Sooronbaev[1], S.J. Singh[2,3]

*Authors contributed equally.

Correspondence to Dr. Maamed Mademilov; mademilov@gmail.com

[1] National Centre of Cardiology and Internal Medicine (NCCIM) named after academician M. Mirrakhimov - Bishkek (Kyrgyzstan)

[2] Department of Respiratory Sciences, University of Leicester (United Kingdom)

[3] Centre for Exercise and Rehabilitation Science, NIHR Leicester Biomedical Research Centre – Respiratory, University Hospitals of Leicester NHS Trust - Leicester (United Kingdom)

[4] Faculty of Health, University of Plymouth (United Kingdom)

[5] School of Sport, Exercise and Health Sciences, Loughborough University (United Kingdom)

Abstract

Objective

After experiencing TB, many people develop post-tuberculosis lung disease (PTBLD). Pulmonary rehabilitation (PR) centrally comprising of education and exercise is recommended internationally for people living with chronic respiratory diseases (CRDs). However, no such service exists in Kyrgyzstan. This study investigated the opinions of healthcare professionals (HCPs) who would be expected to be potential future referrers to PR and adults living with PTBLD about what a PR program could look like in Kyrgyzstan.

Design

A qualitative study using interviews and focus groups. Grounded theory and thematic analysis were used for data collection and analysis.

Participants

63 participants; 15 referrers (12 male, 3 female; 12 pulmonolgists, 3 TB specialists) and 48 adults (26 male, 22 female) living with PTBLD.

For peer review only

Setting

Participants were recruited from hospital settings in Bishkek and Chuy Region, Kygryzstan.

Methods

Fifteen semi-structured interviews were conducted with referrers and 9 focus group discussions were conducted with adults living with PTBLD.

Results

Five key themes were developed; (i) living with PTBLD; (ii) attitude to PR, which emphasized the perceived importance and potential benefits of implemention; (iii) barriers/facilitators to PR, which included time and cost, and the importance of appropriate communication in enabling participation; (iv) interventional components of PR, which described culturally and demographically appropriate physical activities including rhythmic movements, dance, and volleyball; and (v) psychosocial support, which demonstrated the importance of psychological support for patients coping with the effects of stigma.

Conclusions

Potential referrers and adults living with PTBLD expressed their support for the implementation of PR. The culture-specific and population-specific issues highlighted in this work demonstrate the need to address stigma and provide certain types of exercise training/education modules for this specific clinical population. In other respects the currently known attitudes/barriers to PR, identified in Western research, appear to apply. The principles of culturally adapting PR may be helpful for those looking to establish similar clinical services in other LMIC and in Central Asia in particular.

Data availability statement

Data relevant to the study has been included. Further data is available upon reasonable request.

For peer review only

Strengths and limitations of this study

- In LMIC, the primary focus of CRD management is pharmacological treatment, with PR being poorly developed and not widely accessible or recognised.
- It is important to design a PR service informed by both the people it is trying to help and those involved in its future delivery, to ensure its appropriateness and to make it attractive to patients, healthcare workers and policy makers.
- Kyrgyzstan is leading the development of PR among many other post-Soviet countries and in Central Asia, where there is currently an absence of rehabilitation services, and therefore ideas about how to successfully implement PR may have significant implications for other LMIC or low-resource contexts.
- The study was conducted in one geographical region in Kyrgyzstan, therefore the implications reported need to be tested to show applicability to other cultural contexts.

Introduction

Worldwide, 10 million people suffer with tuberculosis (TB) and 1.5 million people die from TB annually. The burden of TB on individuals and society falls predominantly in Low- and Middle-Income Countries (LMIC)[1] where weak economic conditions aggravate the situation of people living with the disease, leading to disability and early mortality[2,3]. Kyrgyzstan is a LMIC in Central Asia with a high poverty level (20.1%)[4,5]. According to the World Health Organisation (WHO), the relative incidence rate of TB in Kyrgyzstan in 2019 was 144 per 100,000, the highest rate in the WHO European Region[6], as well as having one of the highest respiratory mortality rates[7].

Many adults who have experienced TB go on to develop post-tuberculosis lung disease (PTBLD). While chronic respiratory diseases (CRDs) are common in the Kyrgyzstan, tuberculosis is the most challenging lung condition to address, because of public fears about the risk of infection to others, contagion which in turn leads to stigmatization, as evidenced by the issuing of a certificate of full recovery for these patients. People with PTBLD experience decreased exercise tolerance and increased cough, shortness of breath and general weakness. This is often accompanied by feelings of social isolation and depression[8,9]. There is evidence of persistent stigmatization of people living with TB even

For peer review only

after recovery[10,11]; compounding problems such as social exclusion, unemployment, unstable housing and limited access to medical care[10,12,13]. Depression, anxiety and somatic symptom disorders have a particularly negative impact on the standard of living of people in LMIC such as Kyrgyzstan[3,14,15].

Kyrgyzstan declared its independence from the Union of Soviet Socialist Republics (USSR) when it collapsed in 1991. The Soviet model of health care, which was centralized and provided universal free access to medical care, was designed for the prevention and early warning of diseases. During the Soviet era, people living with PTBLD across the USSR attended pulmonary rehabilitation (PR), where services ranged from three weeks to three months and included breathing exercises, prescribed walking, massage and various ball games. In addition, there were other alternative treatment institutions such as sanatoriums, salt mines and alpine resorts.

After the collapse of the Soviet Union, health care systems in independent states have experienced an economic crisis, an outflow of specialists, a decrease in the quality of medical care, a shortage of drugs, and an increase in the number of diseases such as TB and Human Immunodeficiency Virus (HIV). While the rehabilitation centers opened during the Soviet era continued to operate after the independence of Kyrgyzstan, due to a lack of funding, the quality and usefulness of the services provided has reduced[16]. Rehabilitation is rarely available to patients. Consequently, the burden of TB and PTBLD has continued to grow since Soviet times[17]. At present, the treatment process for these individuals is exclusively pharmacological (lasting between 6 months and 2 years) and, with no post-treatment care pathway, they are often left in a state of limbo whereby they are no longer infectious but are living with chronic symptoms and associated stigma. It is important to note that pulmonary rehabilitation is not implemented anywhere in Central Asia.

The importance of PR is well evidenced for people living with CRDs, including for adults living with PTBLD[10,18,19]. PR involves initial assessment followed by individualized therapy (exercise training and education) to improve physical and psychological well-being, and develop a long-term commitment to healthy behavior[20,21]. PR improves shortness of breath, exercise tolerance, and quality of life and reduces the number and duration of hospitalizations linked to exacerbations[18,22,23].

For peer review only

However, the introduction of PR in LMIC needs to be sensitive to both the local populations receiving care and the local context. Access to PR is typically through a referral process whereby healthcare professionals (HCPs) introduce PR to patients. In many LMIC, including Kyrgyzstan, Pulmonologists and TB Specialists work directly with adults living with PTBLD and will likely be involved in referring patients for PR. Consequently, this study combines the views of potential PR referrers and adults living with PTBLD in Kyrgyzstan to identify both the potential acceptability and most appropriate design for PR.

Aims

To explore the views and experiences of potential referrers and adults living with PTBLD in Kyrgyzstan to inform the development of a culturally-appropriate PR service.

Methods

Patient and Public Involvement

As respiratory clinicians, we drew on our experiences of talking to people living with PTBLD when designing the study, in particular the interview schedule and logistics. Interviews and focus groups were organized based on work schedules and family commitments. This work was conducted to provide more in-depth insight into the views of patients to improve the quality of care they receive, such as the provision of appropriate PTBLD-specific PR. Patients were not directly involved in formulating the interview schedules or research question in the traditional sense of patient and public involvement. It was through informal conversations with patients and clinical consultations with patients that the importance of directly involving patients in the design of their PR arose; not least because of the stigma and alienation experienced as a result of their PTBLD. Patients who were involved in the research and resulting PR trial will be invited to support future dissemination activities. By the nature of our qualitative research, the findings of the present study are being used to develop a patient-informed PR programme in accordance with the principles of PPI. The findings of this study will be disseminated to participants and the wider public through awareness-raising activities in TB hospitals to help boost the profile of PR and inform the wider public about PTBLD-specific rehabilitation. The findings will also be disseminated through national and international conferences. The results of this study are expected to

For peer review only

inform the Kyrgyz healthcare system by introducing a culturally-appropriate rehabilitation service informed by patients themselves.

Participants and data collection

In line with the research protocol, HCPs in regular contact with adults living with PTBLD were invited to participate and were recruited from 5 sites in the Chuy region that offer PTBLD treatment (National Hospital, National TB Hospital, City TB Hospital, Chuy region TB Hospital & Alamedin District TB Hospital). A total of 17 HCPs were invited to take part. Two HCPs declined to participate due to lack of time, leaving 15 individuals who participated in semi-structured interviews (12 females, 3 males; 3 Pulmonologists and 12 TB Specialists).

Participants for focus group (FG) discussions were invited from 8 sites (National Hospital, National TB Hospital, NCCIM, City TB Hospital, Chuy region TB Hospital, Sokuluk District TB Hospital, Alamedin District TB Hospital & Kant District TB Hospital). All participants had previously received and finished basic treatment fo PTBLD. In total, 9 FGs were organized. 80 people were invited to participate, of whom 48 took part (26 men and 22 women) in groups of between 4 and 8. Participants came from a range of socio-economic backgrounds (including some homeless participants) and geographical locations (urban and rural).

The sampling strategy was a combination of targeted and convenience sampling. We targeted only medical specialists whose clinical practice directly involved dealing with PTBLD patients. All participants provided written informed consent. The interviews with referrers were conducted face-to-face at the participants' workplace in various health care facilities, both in urban (national center, city center) and rural settings between October 2019 and March 2020. The FGs were conducted on the premises of the department of respiratory medicine of the NCCIM between October 2019 and January 2020. During the FGs, participants were free to interact with each other, offer peer-to-peer support, ask and answer questions, and this method enabled patients to provide extended discussions of topics that were of particular interest to them, such as the problems around stigma. The interviewer (MM) was a Pulmonologist in Bishkek, Kyrgyzstan with previous experience in qualitative research. Interviewer MM acted as facilitator during focus groups. MM did not have any relationship with participants prior to study commencement and equally, participants did not know MM prior to the interviews.

For peer review only

The interview guides were structured using a narrative approach with additional prompts. Different interview guides were used for interviews and focus groups (See interview guides in supplemental appendix 1, 2). To improve and adapt questions and prompts, notes were taken and discussions of transcribed data were regularly held with the research team. The interviews and FGs were conducted in Russian and Kyrgyz languages according to participant preference. The researchers used audio recordings to collect the data. The average interview duration was 30 minutes, and for FGs 50 minutes. There were no follow-up interviews or FGs.

The the interview recordings were transcribed verbatim and then translated into English to enable the cross national team to fully participate in the analysis. The translation was carried out by a Kyrgyz team who were able to understand and interpret all expressions, and conveyed such details as laughter, expressions, etc. in the transcripts. Specialist and local terms were converted into an accessible form for understanding in English. Each interview and FG transcript was assigned a sequence number. Transcripts were anonymized and were not returned to participants for verification.

Data Analysis

The theoretical framework used was grounded theory and the data were analyzed using thematic analysis[24]. The flexibility and explorative character of thematic analysis makes it particularly appropriate for comparing and contrasting qualitative data drawn from different sample groups, and where researchers have variable levels of understanding of the life-worlds of different participant groups.

Each step of the analysis was carried out independently by the Kyrgyz and UK teams, followed by joint discussions, and agreement on the key themes. The first step involved reading and re-reading the transcripts several times (in their original or translated form), and writing a brief summary of the data. Step two involved manually creating codes (software packages were not used). There were seven coders in total; three in Kyrgyzstan, and four in the UK. The combination of similar phrases and sentences were highlighted in the same color, placed under the same code and a description of its contents was developed. A total of 21 initial codes were created. The third step involved refining the themes by grouping together similar codes. The fourth step involved collating all of the

For peer review only

relevant quotes and clarifying how each data extract related to the different themes. The fifth step involved developing a detailed description of themes and subthemes together with the use of relevant quotes.

Summary findings and interpretations encapsulating the perspectives of both referrers and adults living with PTBLD are presented. For the purposes of this article, data have been organized into five overarching themes, impacting a range of different PR-related issues. Quotes were selected from frequently encountered codes during the interviews and FGs and chosen as the best representation of the analytic point. Recruitment targets were determined a priori according to the norms of the respective methods. Partly due to the delays imposed by transcription, translation and cross-national analysis, but also due to the relative ease of access, we recruited up to these targets. Data saturation was achieved, but known only retrospectively as the data analysis progressed.

Results

Referrers and respondents living with PTBLD were positive about the potential introduction of PR. They identified a range of barriers/facilitators that would need to be considered in the design and implementation of any such program. A range of potential PR components were identified, as well as the need to tailor PR to individual physical conditions, provide effective communication and education, and be sensitive to the patients' psychosocial state. It was also noted the importance of including psychological support in the PR, which will be specific to patients with post-TB disease who have stigma and psychological problems. Respondents living with PTBLD shared their illness experiences, such as symptom burden, challenges faced, and patterns of exercise and physical activity, and provided a vision of how a PR program should best be designed. Themes obtained in the thematic analysis are presented in the diagram (See diagram in supplemental appendix 3).

Living with PTBLD

Respondents living with PTBLD identified a range of expected symptoms including, "weakness and shortness of breath" (#FG2). However, the severity of symptoms varies for people living with PTBLD and affects their daily life in different ways. For some, symptoms do not present any difficulties for day-to-day living; "At the moment I am walking freely, doing household chores" (#FG5). But how strongly the respiratory symptoms of PTBLD

For peer review only - http://bmjopen.bmj.com/site/about/guidelines.xhtml

affect quality of life was noted by another respondent: "...with any physical movement I have shortness of breath... shortness of breath is strong" (#FG9).

Many respondents living with PTBLD worry about contracting TB again. They strictly monitor their symptoms and when their condition worsens or new symptoms emerge, they turn to medical staff for advice:

> "...I do everything that the doctor said, every year I go to the clinic to [the] TB specialist, cough, sneeze, get a yearly consultation, every year I go to X-rays" (#FG1)

In addition to recognizing the efficacy of traditional treatments, some respondents resort to alternative treatments; "...I drink rose hips, sometimes with lemon, I eat a spoonful of honey all the time, I try to drink milk, yogurt" (#FG1). Many respondents choose spa treatment: "... I went to the salt room, in the sanatorium, Kyrgyz seaside" (#FG1). Others noted that movement and physical exercise in general improved their well-being:

> "I...try to walk, sometimes I walk from morning to evening. I believe that walking, in general, physical activity is a huge benefit" (#FG5)

Particularly in rural communities, where agricultural work is common, day-to-day living is relatively active. Others noted how activity was embedded into their lifestyle - "I walk a lot, do not use the washing machine, cook and wash" (#FG5) – or did certain activities at home: "...I do breathing exercises so as not to cough. In these moments, gymnastics helps me" (#FG2).

Respondents have become accustomed to living with respiratory symptoms; "I wanted to live and breathe deeply, we have already forgotten what it is, but we want it so much" (#FG1). They are frequently proactive, seeking to improve their condition; "...we make different attempts, we want to get better. We need life" (#FG2). Consequently there is a significant demand for the implementation of a service like PR, which can help improve health-related quality of living.

Attitude to PR

All referrers noted the importance of implementing PR and recognized that the current absence of services meant that, post-treatment, people living with PTBLD were left without effective guidance:

For peer review only

> "Rehabilitation is the process necessary after completion of treatment for complete recovery" (#R12)

This sense of importance was confirmed by the FG data, whereby participants noted the potential of PR for improving their quality of life, increasing exercise tolerance and reducing the severity of symptoms: "If I constantly move, I feel good, nothing hurts" (#FG8). However, some have a limited awareness about which types of activities are suitable - "…you can or cannot play sports…, I don't know…" (#FG4) - or how to participate in these activities safely; "…we don't know what movements to do and how to do them correctly" (#FG2).

Consequently, FG respondents noted the importance of receiving trustworthy, expert advice about PR and qualified assistance from medical personnel; "health professionals need to be trained so that I can come and ask, and not receive the answers that I know" (#FG1). HCPs should also adapt PR to individual needs: "The main thing is that the coach is good, that he knows how to give the right load" (#FG5).

Referrers noted the value of helping people to improve physical function and adapt to social life. As one referrer noted, PR would:

> "[lead to] an increase in tolerance to physical exertion, it would be like an addition to the main treatment… then it would reduce the number of complications, the number of exacerbations in general... so that the diseases do not become more complicated, do not worsen, as it seems to me that PR has a huge role [to play]." (#R9)

Respondents living with PTBLD generally exhibited a positive attitude towards PR with many expressing an interest in participating: "I will gladly come" (#FG1). Another respondent stated: "Yes, absolutely necessary" (#FG3). The FGs also revealed that some people might not want to take part in PR; for example, people who believe they are relatively healthy or have mild symptoms may be uninterested and see no benefit in participating.

Barriers/Facilitators to PR

HCPs identified relatively few barriers to referring patients to PR. Only one fundamental issue was identified as precluding PR participation; namely the presence of concomitant diseases (#R11) or unstable conditions (#R9).

For peer review only

Referrers did however note that some personality types – "phlegmatic people" (#R8), those whose behavior was erratic, "forgetful, shy" (#R11) - were not well suited to PR and that this would affect uptake. The importance of personality type was also noted by FG respondents:

> "Among the patients there are aggressive patients who run into others for no reason, and there are calm ones on the contrary, who prefer more privacy" (#FG7)

A further consideration was the social situation of some adults. It was noted that many migrate (primarily for economic reasons) after finishing treatment. It was also noted that, "among the patients with TB, we have former convicts and drug addicts" (#R10), "alcoholics, homeless people" (#R4) and so PR opportunities would not be taken up by everyone.

More generally, cost and time were identified as major considerations. A number of referrers argued that PR must be free to encourage attendance and meet the expectations of those living with PTBLD (#R5, 11, 13). FG respondents felt the same (#FG1) and emphasized the potential financial burden of implementing PR: "I wish there was a discount. Many patients do not work, there is little money…"(#FG2).

Personality types and the social situation were identified as interdependent factors. One referrer noted that attitudes from those with PTBLD were affected by the experience of having TB; "…because our patients have been treated for a long time, they are asocial for a very long time" (#R1). Others felt that the prolonged treatment period often experienced would make people reluctant to volunteer for further engagement. Some referrers noted that adherence could be affected by slow positive effects on health, which can lead to a loss of interest:

> "…they don't feel [the effects] right away… gradually if this is repeated regularly, you will feel a positive effect" (#R2)

The two primary solutions proposed for these issues were educating the patient with a "conversation about the need for post-treatment rehabilitation" (#R1), and attending to the "dynamics" and "subjective feelings" (#R2) generated in the PR environment that will help strengthen adherence to PR.

Referrers talked about the need for effective doctor-patient communication for the success of PR. In the context of referring a patient to PR, interviewees noted that the authority and

For peer review only

recommendations of the attending physician will have a very important impact on a person's decision to participate in PR:

> "If you explain normally to a person, any person will do what the doctor recommends, because in our society, as a doctor, as long as we have authority, patients believe us." (#R3)

Most respondents noted that they would follow recommendations to improve their health:

> "You need to do whatever your doctors tell you, take the right medication, eat right" (#FG3).

However, positive clinician-patient relations cannot be taken for granted as some of the FG respondents recalled unfavourable experiences with medical staff in the past. Being open and friendly was seen as an important source of motivation for those living with PTBLD;

> "…if you communicate openly with them, they are also open patients, and they will be happy to hear from you… happy to know any information." (#R2)

However, this alone would not be sufficient to mitigate experiences of stigma, which created a closed-minded attitude amongst those affected. Referrers explained how this could affect interactions with other HCPs:

> "…because even if we write a certificate for them, … [that] they are not contagious to others, sometimes in the consultation department or even in the hospital they look at patients differently and say 'you are ill with TB'" (#R12)

Interventional components of PR

While all referrers and FG respondents noted the importance of implementing physical exercises, a broad range of specific exercises were described. Referrers noted that walking was "the simplest and cheapest method" (#R2), while swimming and Nordic walking were commended for activating a wide range of muscles (#R9, #R15). Other activities included rhythmic movements (dancing, wushu [Chinese Kungfu]), ball sports (volleyball, basketball) and recreational activities (running, cycling, gymnastics). Like many countries, Kyrgyz culture is heavily influenced by dance, and the national dance "Kara Jorgo" ("Black Stallion") was cited as an activity that may be suitable to include in PR (#R12). Referrers argued for

For peer review only

"...almost all the exercises that involve the respiratory muscles, [and] the muscles of the shoulder girdle ... especially the muscles of the arms and shoulders" (#R9). They further noted the "...need to try everything..." (#R5) because different people like different forms of exercise.

Referrers and FG respondents also supported singing as part of the rehabilitation, though there were several opposing opinions. For instance, FG participants argued that "singing songs can be added to rehabilitation... it's good for the lungs" (#FG5), while referrers noted that attention should be paid to contraindications and severe concomitant diseases, which can lead to serious consequences such as internal bleeding:

> "If they have changes in the lungs, if they have caverns, then... singing can somehow affect the deterioration of their condition" (#R1)

But of greater concern to the referrers and FG respondents was that the intensity of activity should be suitable to the individual. Exercises needed to be done, "...not at such a pace, but ... sparing, light" (#R4). As one FG respondent remarked, "...don't overwork" (#FG4). Referrers rejected "...the dances where they jump intensively there, run there," (#R9) and recommended activities such as:

> "...rhythmic dances, then slow ... they can still use the respiratory muscles in their work and [this] may, it seems to me, be useful for recovery" (#R9)

Additionally, FG respondents emphasized the importance of developing the correct schedule and suggested having both morning and evening classes because, "sometimes they [people] are not allowed to leave work" (#FG1). Others noted that the time pressures experienced by those who work or have domestic or childcare duties meant that it would be good if "some exercises can be done at home" (#R11).

Finally, referrers stated that where education was offered to those with PTBLD, verbal delivery was preferable to reading or text:

> "...video lectures should be organized in all clinical specialized pulmonary units. They should be concise, lasting 10-15 minutes" (#R6)

Despite various challenges, the strength of desire to overcome the consequences of PTBLD was evident in the respondents' willingness to spend their limited time and money to

For peer review only

participate in PR (#FG1). The benefits of PR were highlighted: "the time spent on recovery is not in vain. To do this, you can postpone other activities" (#FG5).

Psychosocial support

All TB specialists noted the need to address psychological problems (e.g. depression, becoming withdrawn or aggressive), personal and relational issues (e.g. with family) and to help people living with PTBLD adapt to social life. According to referrers, people may experience stigmatization from a variety of sources. Some people "leave the family" (#R13) due to the stigma of illness, while others are afraid that they will meet people they know at the hospital; "…[patients] tell us where and in which hospitals their relatives, friends work and do not want to visit [these places]' (#R15). Another noted that, "stigma discrimination is very common among our patients, and I think that psychosocial support can be connected to PR" (#R2).

This was supported by the FG data, where people shared their experiences of PTBLD-related stigma. Several participants said they experienced difficulties finding a job; "I tried so many times to get a job, they didn't take me" (#FG7). Some people struggle to recover and after treatment and discharge, they are subjected to stereotyping, affecting their relationships with their loved ones. For example,

> "My neighbor...told her (daughter-in-law) and the children not to come to me, that I was contagious, and to other neighbors that I needed to be evicted." (#FG3)

Indicatively, it was suggested PR should be done "anonymously so that no one knows" (#FG1).

Consequently, both referrers and FG participants placed significant emphasis on the role of psychological support within PR interventions. It was argued that some psychological support would be provided through the social nature of some PR activities. Where rehabilitation takes place in a group, all adults with PTBLD "will be equal … to each other, they will talk, they will communicate" (#R3). Conducting group PR discussions could provide mutual support because, "…until a person gets sick, they do not understand others, and there everyone understands each other, they talk, they share secrets" (#R11). There was also a call for more formal, professional psychological support; "we need psychological therapy" (#FG7).

For peer review only

Thus, it was not only argued that PR needed to incorporate psychological support but that it offers a good opportunity to provide the kind of support that was more generally needed by people living with PTBLD. As one FG respondent noted, "The main thing is psychological support. Sport is sport, but warm words cannot be replaced by anything" (#FG5).

Discussion

The need to develop appropriate PR services for people living with PTBLD, and who reside in LMIC, is an important issue that has scarcely been studied. Kyrgyzstan is leading the development of PR among many other post-Soviet countries and in Central Asia, where there is currently an absence of rehabilitation services. Consequently, about how to successfully implement PR may have significant implications for other LMIC or low-resource contexts. This qualitative study synthesized the views of adults living with PTBLD, and the TB Specialists and Pulmonologists who would initiate referral, to explore how PR could be tailored to maximize uptake in the context of Kyrgyzstan. The results indicate both a general need and willingness to embrace PR, as well as key barriers (e.g., cost, time) and facilitators (e.g. staffing, scheduling, and delivery mode) to uptake, and specific guidance on the components of intervention, including the role of psychosocial support. PR was viewed as playing an important role in improving both people’s physical and mental health.

The integration of PR into the treatment pathway should be supported by strong long-term relationships between patients and clinicians, primarily a TB specialist or Pulmonologist. The data suggest that a general inclination to respect the authority of medical professionals, and a widespread compliance with treatment recommendations, will be enhanced by an open and honest attitude towards patients. Good communication by HCPs is needed to build rapport and trust with a cohort who may have experienced discrimination. There is a need to include PR in doctor-patient conversations early in the TB treatment process so that those who go on to develop PTBLD are aware that PR is a necessary and valuable part of their overall recovery and reintegration into society. Because of the contrasting medical guidance at the treatment and rehabilitation phases for TB (i.e. initially rest and latterly activity), clinicians should be transparent and consistent in promoting awareness of the benefits of PR early on during treatment. This will likely boost the uptake to PR.

For peer review only

In particular, continued patient engagement will be influenced by social factors. Because social attitudes prevent some patients from participating in hospital-based PR services, it is vital to design a recruitment pathway and appointment logistics that are sensitive to issues relating to stigmatization. For example, it may be suitable to conduct PR classes at quieter times of the day, to help patients arrange transport or to offer overnight accommodation for those travelling far from rural regions. It would be desirable to provide advice about exercise that can be done at home and particularly about what level of exertion is both beneficial and safe. Many people living with PTBLD experience financial difficulties[25], and either struggle to find employment or secure only relatively low-paid jobs. Therefore, when organizing PR for such a population, discounted or low-cost provision is essential. It is significant that participants preferred modes of exercise training that do not require expensive equipment and could be undertaken in home or community settings, such as walking, dance and balls sports as this facilitates the implementation of PR in LMIC.

Educational components of PR should be tailored to the needs of the particular audience. In parts of the world where PR is part of standard care, education has traditionally been delivered in a lecture style but the data suggest that information provided verbally, with supporting images, and in ‘bite size’ amounts, would be more suitable for the PTBLD population in Kyrgyzstan. This fits with an ongoing transformation of PR education delivery in other parts of the world, such as in the UK, to move away from didactic deliveries, with the educational and cultural backgrounds of PR attendees considered[26].

Many previous studies have focused on stigma related to the infectious nature of TB and the lack of awareness within the general population that these individuals are no longer contagious after completing their drug treatment[11]. Studies conducted in LMIC such as Vietnam[27] and India[11], reported that TB is often deemed a ‘death penalty’ and perceived as a ‘dirty disease’. Studies in LMIC have shown that the effects of stigma can both influence a patient’s treatment and care-seeking behavior[28,29] and undermine patients’ trust in HCPs[30]. A previous Kyrgyzstan study found that TB patients frequently experience discrimination from HCPs who sought to avoid interaction[15].

A major lesson from this study is that individuals with PTBLD may continue to experience discrimination, including from HCPs who deny them advice and make it difficult for them to visit healthcare facilities. PR offers an opportunity for these individuals to receive care from

For peer review only

HCPs free from prejudice. Incorporating educational sessions will help them understand their disease and the social attitudes they experience. In this regard, both sets of respondents emphasized the importance of incorporating psychosocial support as part of PR to help people cope with the effects of stigma. Incorporating group sessions in a safe, non-judgmental environment offers peer-peer support and will enable patients to share their experiences openly.

Another way to promote PR attendance and adherence is to embed culturally and demographically-appropriate activities. The core PR components of aerobic exercise typically include walking/cycling, strength training and education[18]. The results of this study suggest that the generic principles of PR can be adapted to the local context or population. For example, respondents spoke about the popularity of ball sports such as volleyball, reminiscent of the rehabilitation offered in the Soviet era. Conventional exercises like squats can be ‘gamified’ into a volleyball dig action to increase enjoyment and motivation. Adaptations are likely to be most successful where they also help patients’ understanding of how these exercises can translate into benefits in daily life e.g. taking part in social activities or preparing for employment.

Respondents supported the incorporation of rhythmic movements such as dance performed at an appropriate intensity. A national dance such as Kara Jorgo, can both be scaled to each person’s physical condition and offer a culturally relevant and enjoyable form of exercise to strengthen muscles involved in the mechanics of breathing. Dance has been shown to improve health, including physical performance and mood[31,32]. Taking part in socially conventional exercises will challenge stigma and show people living with PTBLD how PR is also relevant to everyday living.

In addition to dancing, singing is also an important part of the Kyrgyz culture. Unlike dancing, singing had a mixed reception from participants some of whom were enthusiastic and some of whom said it would not be popular[33]. Although singing therapy has been tested previously, with evidence suggesting that it can improve wellbeing for adults with COPD[34,35], evidence relating to the effects of singing for PTBLD populations is lacking. Consequently, further research is required before implementing singing as part of PR in the contexts of PTBLD and Central Asia.

For peer review only

Limitations

The study was conducted in one geographical region in Kyrgyzstan, therefore the applicability of the implications reported require empirical verification. The study focused on PR for PTBLD rather than the broader spectrum of lung diseases, but it is important to note that there is no evidence to suggest that the implementation of PR for PTBLD is likely to be easier in relation to this group, due to the stigma associated with the condition.

Conclusion

PR is embedded in routine clinical care in Western countires with unequivocal evidence of its benefits. In this qualitative exploration, potential PR referrers (TB Specialists and Pulmonologists), as well as adults living with PTBLD, were overwhelmingly positive towards the implementation of PR in Kyrgyzstan. The culture-specific and population-specific issues highlighted in this work demonstrate the need to address stigma and provide certain types of exercise training/education modules for this specific clinical population. In other respects the currently known attitudes/barriers to PR, identified in Western research, appear to apply. The act of culturally adapting PR may be beneficial for other settings looking to establish PR as a clinical service or evaluate PR in clinical trials in low resource settings. The results of this study can be applied to provide a basis for the implementation of PR programs and to evaluate the effectiveness of such programs for people with PTBLD. Future research should focus on the geographical generalizability of these findings, and the applicability for PR programs for other CRDs.

Footnotes

Ethics statements

Ethics approval

This work is part of NIHR Global Health Research Group on Respiratory Rehabilitation (Global RECHARGE; 17/63/20), approved by The National Center for Cardiology and Internal Medicine (NCCIM; ref: 17) and University of Leicester (ref: 22293) ethics committees. This study was registered prospectively (ISRCTN16501214).

Funding: This research was funded by the National Institute for Health Research (NIHR) (17/63/20) using UK aid from the UK Government to support global health research.

For peer review only

Acknowledgments

Competing Interest statement

All authors declare no competing interest.

Contributorship statement

M. Mademilov - conducting interviews (data collection), thematic analysis, data interpretation and writing an article

G. Mirzalieva - conducting interviews (data collection), supporting thematic analysis, data interpretation and writing an article

Z.K. Yusuf - supporting thematic analysis, data interpretation and writing an article

M.W. Orme - supporting thematic analysis, data interpretation and writing an article

C.L.A. Bourne - supporting thematic analysis and writing an article

A. Akylbekov - supporting thematic analysis and writing an article

A.V. Jones - assisting with interview scheduling and data interpretation

R.B. Miah - assisting with interview scheduling and data interpretation

R. Jones - supporting with data interpretation and data analysis

A. Barton - supporting thematic analysis and writing an article

D. Malcolm - supporting thematic analysis, data interpretation and writing an article

T. Sooronbaev - development of the concept and design of the study and writing an article

S.J. Singh - development of the concept and design of the study and writing an article

Data availability statement

Data are available upon reasonable request

For peer review only

References

1. Tuberculosis. *World Health Organization*, World Health Organization, 2019, www.who.int/health-topics/tuberculosis#tab=tab_1.

2. Jaramillo, E. Pulmonary tuberculosis and health-seeking behaviour: how to get a delayed diagnosis in Cali, Colombia. *Tropical medicine & international health: Trop. Med. Int. Heal.* (1998); 3.2: 138-144.

3. Peltzer, K. *et al.* Prevalence of psychological distress and associated factors in tuberculosis patients in public primary care clinics in South Africa. *BMC psychiatry* (2012);12.1: 1-9.

4. About Us: UNDP in Kyrgyz Republic. *UNDP*, 2015, www.kg.undp.org/content/kyrgyzstan/en/home/about-us.html#:~:text=Kyrgyzstan is a landlocked%2C largely,income of %241%2C170 in 2015.

5. Living standard. *National Statistical Committee of the Kyrgyz Republic*, 2019. http://www.stat.kg/en/statistics/uroven-zhizni-naseleniya/.

6. Tuberculosis Surveillance and Monitoring in Europe, 2019. *European Centre for Disease Prevention and Control*, www.ecdc.europa.eu/en/publications-data/tuberculosis-surveillance-and-monitoring-europe-2019.

7. Gibson, J., *et al.* The Burden of Lung Disease. *ERS*, 2013, www.erswhitebook.org/chapters/the-burden-of-lung-disease/.

8. Muñoz-Torrico, M. *et al.*, Is there a rationale for pulmonary rehabilitation following successful chemotherapy for tuberculosis?, *J. Bras. Pneumol.* (2016); 42.5: 374–385, 2016.

9. Cheng-Ting, L. *et al.* Evaluation of health-related quality of life in patients with tuberculosis who completed treatment in Kiribati. *Journal of International Medical Research* (2017); 45. 2: 610-620.

10. Allwood, B.W *et al.*, Post-tuberculosis lung health: Perspectives from the First International Symposium. *Int. J. Tuberc. Lung Dis.* (2020); 24. 8: 820–828.

11. Rajeswari, R. *et al.* Perceptions of tuberculosis patients about their physical, mental and social well-being: a field report from south India. *Soc. Sci. Med.* (2005); 60. 8: 1845–1853.

12. Datiko, D.G., Jerene, D., and Suarez, P. Stigma matters in ending tuberculosis: Nationwide survey of stigma in Ethiopia. *BMC Public Health* (2020); 20. 1: 1–10.

13. Bauer, M. *et al.*, Health-related quality of life and tuberculosis: A longitudinal cohort study. *Health Qual. Life Outcomes* (2015); 13.1: 1–13.

14. Walker, I. F., *et al.* Implementation of a psychosocial support package for people receiving treatment for multidrug-resistant tuberculosis in Nepal: A feasibility and acceptability study. *PloS one* (2018); 13.7: e0201163.

15. Burtscher, D. et al. 'My favourite day is sunday': Community perceptions of (drug-resistant) tuberculosis and ambulatory tuberculosis care in Kara Suu District, Osh Province, Kyrgyzstan. *PLoS One*, (2016); 11. 3: 1–16.

16. Rechel, B. Health policy reform in the countries of the former Soviet Union. *The Palgrave International Handbook of Healthcare Policy and Governance*. Palgrave Macmillan, 2015.

17. Arinaminpathy, N. and Dye, C. Health in financial crises: Economic recession and tuberculosis in Central and Eastern Europe. *J. R. Soc. Interface* (2010): 7. 52: 1559–1569, 2010.

18. McCarthy, B. *et al*. Pulmonary rehabilitation for chronic obstructive pulmonary disease. *Cochrane database Syst. Rev.* (2015); 2: CD003793.

19. Singh, S.K., *et al.* Pulmonary Rehabilitation in Patients with Chronic Lung Impairment from Pulmonary Tuberculosis. *Cureus* (2018); 10. 11.

20. Jones, R. *et al*. Does pulmonary rehabilitation alter patients' experiences of living with chronic respiratory disease? A qualitative study. *Int. J. COPD* (2018); 13: 2375–2385.

21. Corhay, J.L., *et al.* Pulmonary rehabilitation and copd: Providing patients a good environment for optimizing therapy. *Int. J. COPD* (2013); 9: 27–39.

22. Griffiths, T.L., *et al.*, Results at 1 year of outpatient multidisciplinary pulmonary rehabilitation: a randomised controlled trial. *Lancet* (2000); 355. 9201: 362–368.

23. Puhan, M.A. *et al*. Pulmonary rehabilitation following exacerbations of chronic obstructive pulmonary disease. *Cochrane Database Syst. Rev.* (2016); 12.

24. Braun, V., and Clarke, V. Using thematic analysis in psychology. *Qualitative research in psychology* (2006); 3. 2: 77-101.

25. Tanimura, T. *et al.* Financial burden for tuberculosis patients in low-and middle-income countries: a systematic review. *European Respiratory Journal* (2014); 43.6: 1763-1775.

26. Bolton, C.E., *et al.* British Thoracic Society guideline on pulmonary rehabilitation in adults: accredited by NICE. *Thorax* (2013); 68. 2: 1-30.

27. Hoa, N.P., *et al.* Knowledge of tuberculosis and associated health-seeking behaviour among rural Vietnamese adults with a cough for at least three weeks. *Scandinavian Journal of Public Health* 2003; 31. 62: 59-65.

28. Ngamvithayapong, J., Winkvist, A. and Diwan, V.. High AIDS awareness may cause tuberculosis patient delay: results from an HIV epidemic area, Thailand. *Aids* (2000); 14. 10: 1413-1419.

29. Weiss, M.G., Ramakrishna, J. and Somma, D. Health-related stigma: rethinking concepts and interventions. *Psychology, health & medicine* (2006); 11.3: 277-287.

30. Miller J A. Perception and beliefs of health care workers about clients with tuberculosis. Auckland: University of Auckland; 2007.

31. Koch, S.C., *et al.* Effects of dance movement therapy and dance on health-related psychological outcomes. A meta-analysis update. *Frontiers in psychology*, (2019); 10: 1806.

32. Williams, S. Dance group enriches lives of people living with breathlessness. *The Lancet Respiratory Medicine* (2020); 8. 8: 763-764.

33. McNamara RJ *et al.* Singing for adults with chronic obstructive pulmonary disease (COPD). *Cochrane Database Syst Rev.* (2017); 12.

34. Lord, V.M., *et al.* Singing classes for chronic obstructive pulmonary disease: a randomized controlled trial. *BMC Pulmonary Medicine* (2014); 14: 181-181.

35. Downes, C. *et al.* Singing for Breathing Uganda: Group singing for people with chronic lung disease in Kampala. *Journal of Applied Arts & Health* (2019); 10. 2: 219-228.

For peer review only

Work Package 1 Patient Focus Group Schedule

The impact has PTBLD has had on their life

- When were you first diagnosed? What was it like to hear that?
- How did you expect PTBLD was going to affect you? Is there anything you wish you had been told about PTBLD? If so, what? Why? How would this have helped you?
- Did you know anyone else who had PTBLD? How are they treated by other people?
- How do you think other people have reacted to you having PTBLD? Did you tell family/friend/work colleagues? Immediately or not?
- What has most surprised you about having PTBLD? What are the differences between what people generally think about PTBLD and the actual experience of having it?
- Consider the impact on your work (employment), your domestic tasks, and leisure choices. What has been the most significant? What? It what ways? Give examples.
- What changes, if any, have you had to make to your life because of PTBLD? Prompt: employment, domestic duties, leisure activities, personal relationships?
- How would you advise others living with PTBLD?
- How do you feel about the future?

Everyday activities:

- When, in your everyday life are you most active?
- Do you prefer individual or social activities when you are being active? Why?
- Are there any activities that you would like to do? Or any that you no longer do that you would like to be able to do again?
- Other participants have talked about activities such as ball sports (e.g., basketball, volleyball, football), wushu, dancing, singing and music – what do you think about doing these activities?

Interviewer: Provide a brief overview of PR: "PR consists of an exercise and education programme. Treatment is tailored to each person and it aims to help you control the symptoms of your condition, educate you about your condition, improve general health and level of fitness, improve your ability to carry out daily activities that are important to you and improve your confidence and quality of life."

- What are your initial thoughts?
- How would you hope PR might help you? What would you hope to achieve from taking part?
- What do we need to think about in making you more comfortable to attend PR?
- Would you be sacrificing anything to be part of this? Time? Money? Energy that will stop you doing other things? What are the negative sides of doing PR?
- What do you think should be included in PR? Why? (PROMPT: psychological support, education, information, types of exercises). **Note: ask them for specific examples**
- Are there any activities or hobbies that could be useful in PR? (PROMPT: ball sports such as basketball, volleyball, football; wushu; dancing; singing and music
- What do you think the challenges might be with these activities as part of PR?

For peer review only

Work Package 2 Referrer Interview Schedule

Aim 1) General opinions about pulmonary rehabilitation

- What does the term pulmonary rehabilitation mean to you?
- What is your opinion about pulmonary rehabilitation?

  [Probing question: How important do you think it is to provide pulmonary rehabilitation?]
- Do you have any experience in rehabilitation?

  If so: What did the rehabilitation consist of?

  How long was the average course of rehabilitation?

  Roughly what percentage of patients would take part in the rehabilitation?
- What would you think about being involved in referring patients to pulmonary rehabilitation?
- What do you think about using your clinical time for pulmonary rehabilitation?
- What do you think the problems will be to referring patients to pulmonary rehabilitation?
- How do you think these problems can be resolved?

  [Probing question: What will be your reasons for referring patients to pulmonary rehabilitation?]
- How would you describe pulmonary rehabilitation to your patients?
- What characteristics will you look for when deciding whether or not to refer a patient?

  [Probing question: Are there certain types of patients you wouldn't even discuss pulmonary rehabilitation with? Why?]
- How do you know if someone is willing or ready to take part in pulmonary rehabilitation?
- Who do you think should be involved in assessing which pulmonary rehabilitation activities would help a specific patient? And why?
- What staff should be involved in providing pulmonary rehabilitation?

For peer review only

Aim 2) Patients' opinion of pulmonary rehabilitation

- How do you think patients will respond to your referral for pulmonary rehabilitation? Why?
- How do you think patients will get on with pulmonary rehabilitation? Why?
- What can we do to help patients complete their course of pulmonary rehabilitation?

Aim 3) Changes needed for pulmonary rehabilitation to work well in Kyrgyzstan

- What physical activities/exercise are good for these patients? Why? How might we do these activities as part of pulmonary rehabilitation?
- What do you think should be included in pulmonary rehabilitation? Why?

  [PROMPT: Education, information, types of exercises (e.g., walking, ball sports, cycling, yoga), psychological assistance, music]

  [Probing question: Why? Why not other activities?]
- What problems are caused by the stigma associated with PTBLD?

  [Probing question: How might these problems impact participation in pulmonary rehabilitation? What can we do to help patients take part? How can we help them to have a positive experience?]

  [PROMPT: environment, social influence, emotional conditions, feeling comfortable]
- Should pulmonary rehabilitation address problems about stigma?

  [Probing question: How might pulmonary rehabilitation reduce stigma?]

For peer review only

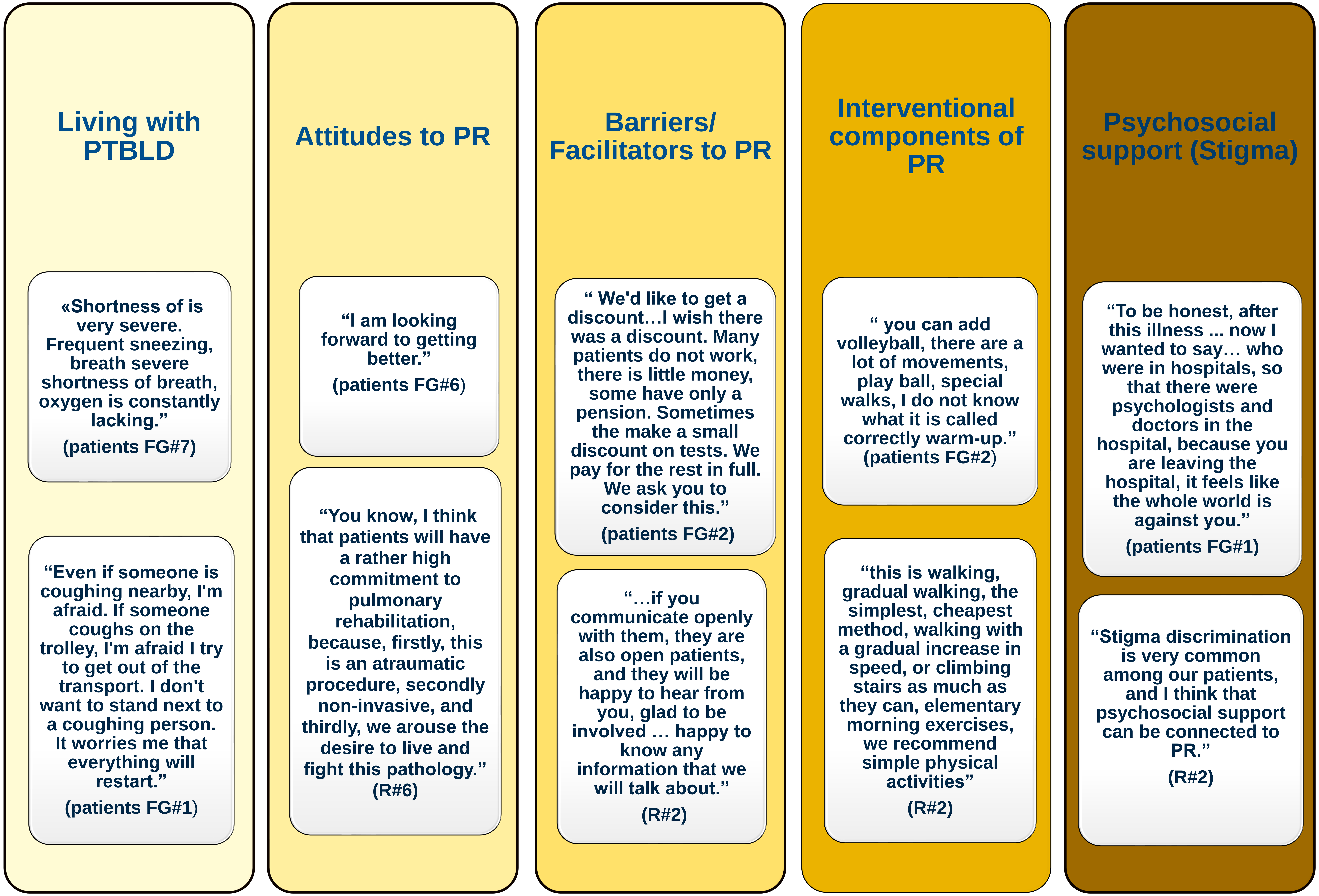
Living with PTBLD
«Shortness of is very severe. Frequent sneezing, breath severe shortness of breath, oxygen is constantly lacking.”
(patients FG#7)
“Even if someone is coughing nearby, I'm afraid. If someone coughs on the trolley, I'm afraid I try to get out of the transport. I don't want to stand next to a coughing person. It worries me that everything will restart.”
(patients FG#1)
Attitudes to PR
“I am looking forward to getting better.”
(patients FG#6)
“You know, I think that patients will have a rather high commitment to pulmonary rehabilitation, because, firstly, this is an atraumatic procedure, secondly non-invasive, and thirdly, we arouse the desire to live and fight this pathology.” (R#6)
Barriers/ Facilitators to PR
“ We'd like to get a discount…I wish there was a discount. Many patients do not work, there is little money, some have only a pension. Sometimes the make a small discount on tests. We pay for the rest in full. We ask you to consider this.”
(patients FG#2)
“…if you communicate openly with them, they are also open patients, and they will be happy to hear from you, glad to be involved … happy to know any information that we will talk about.”
(R#2)
Interventional components of PR
“ you can add volleyball, there are a lot of movements, play ball, special walks, I do not know what it is called correctly warm-up.” (patients FG#2)
“this is walking, gradual walking, the simplest, cheapest method, walking with a gradual increase in speed, or climbing stairs as much as they can, elementary morning exercises, we recommend simple physical activities”
(R#2)
Psychosocial support (Stigma)
“To be honest, after this illness ... now I wanted to say… who were in hospitals, so that there were psychologists and doctors in the hospital, because you are leaving the hospital, it feels like the whole world is against you.”
(patients FG#1)
“Stigma discrimination is very common among our patients, and I think that psychosocial support can be connected to PR.”
(R#2)

BMJ Open

What should pulmonary rehabilitation look like for people living with post-tuberculosis lung disease in the Bishkek and Chui region of the Kyrgyz Republic?: A qualitative exploration

Journal:	*BMJ Open*
Manuscript ID	bmjopen-2021-053085.R2
Article Type:	Original research
Date Submitted by the Author:	28-Dec-2021
Complete List of Authors:	Mademilov, Maamed; National Center for Cardiology and Therapy named after academician Mirsaid Mirrakhimov under the Ministry of Health of the Kyrgyz Republic, Respiratory medicine<br>Mirzalieva, Gulzada; National Center for Cardiology and Therapy named after academician Mirsaid Mirrakhimov under the Ministry of Health of the Kyrgyz Republic, Respiratory medicine<br>Yusuf, Zainab; University of Leicester Department of Respiratory Sciences, Department of Respiratory Sciences ; University Hospitals of Leicester NHS Trust, Centre for Exercise and Rehabilitation Science, NIHR Leicester Biomedical Research Centre – Respiratory, University Hospitals of Leicester NHS Trust<br>Orme, Mark; University of Leicester, Department of Respiratory Sciences; University Hospitals of Leicester NHS Trust, Centre for Exercise and Rehabilitation Science, NIHR Leicester Biomedical Research Centre – Respiratory<br>Bourne, Claire; University Hospitals of Leicester NHS Trust, Centre for Exercise and Rehabilitation Science, NIHR Leicester Biomedical Research Centre – Respiratory<br>Akylbekov, Azamat; National Center for Cardiology and Therapy named after academician Mirsaid Mirrakhimov under the Ministry of Health of the Kyrgyz Republic, Respiratory medicine<br>Jones, Amy; University of Leicester, Department of Respiratory Sciences; University Hospitals of Leicester NHS Trust, Centre for Exercise and Rehabilitation Science, NIHR Leicester Biomedical Research Centre – Respiratory<br>Miah, Ruhme ; University of Leicester, Department of Respiratory Sciences; University Hospitals of Leicester NHS Trust, Centre for Exercise and Rehabilitation Science, NIHR Leicester Biomedical Research Centre – Respiratory<br>Jones, Rupert; University of Plymouth, Faculty of Health<br>Barton, Andy; University of Leicester, Department of Respiratory Sciences<br>Malcolm, Dominic; Loughborough University, School of Sport, Exercise and Health Sciences<br>Sooronbaev, Talant; National Center for Cardiology and Therapy named after academician Mirsaid Mirrakhimov under the Ministry of Health of the Kyrgyz Republic, Respiratory medicine<br>Singh, Sally; University of Leicester, Department of Respiratory

	Sciences; University Hospitals of Leicester NHS Trust, Centre for Exercise and Rehabilitation Science, NIHR Leicester Biomedical Research Centre – Respiratory
**Primary Subject Heading**:	Rehabilitation medicine
Secondary Subject Heading:	Respiratory medicine
Keywords:	Tuberculosis < INFECTIOUS DISEASES, Respiratory infections < THORACIC MEDICINE, REHABILITATION MEDICINE, RESPIRATORY MEDICINE (see Thoracic Medicine), QUALITATIVE RESEARCH

SCHOLARONE™
Manuscripts

I, the Submitting Author has the right to grant and does grant on behalf of all authors of the Work (as defined in the below author licence), an exclusive licence and/or a non-exclusive licence for contributions from authors who are: i) UK Crown employees; ii) where BMJ has agreed a CC-BY licence shall apply, and/or iii) in accordance with the terms applicable for US Federal Government officers or employees acting as part of their official duties; on a worldwide, perpetual, irrevocable, royalty-free basis to BMJ Publishing Group Ltd ("BMJ") its licensees and where the relevant Journal is co-owned by BMJ to the co-owners of the Journal, to publish the Work in this journal and any other BMJ products and to exploit all rights, as set out in our licence.

The Submitting Author accepts and understands that any supply made under these terms is made by BMJ to the Submitting Author unless you are acting as an employee on behalf of your employer or a postgraduate student of an affiliated institution which is paying any applicable article publishing charge ("APC") for Open Access articles. Where the Submitting Author wishes to make the Work available on an Open Access basis (and intends to pay the relevant APC), the terms of reuse of such Open Access shall be governed by a Creative Commons licence – details of these licences and which Creative Commons licence will apply to this Work are set out in our licence referred to above.

Other than as permitted in any relevant BMJ Author's Self Archiving Policies, I confirm this Work has not been accepted for publication elsewhere, is not being considered for publication elsewhere and does not duplicate material already published. I confirm all authors consent to publication of this Work and authorise the granting of this licence.

For peer review only

What should pulmonary rehabilitation look like for people living with post-tuberculosis lung disease in the Bishkek and Chui region of the Kyrgyz Republic?: A qualitative exploration

Author affiliations

M. Mademilov[1]*, G. Mirzalieva[1]*, Z.K. Yusuf[2,3], M.W. Orme[2,3], C.L.A. Bourne[3], A. Akylbekov[1], A.V. Jones[2,3], R.B. Miah[2,3], R. Jones[4], A. Barton[2], D. Malcolm[5], T. Sooronbaev[1], S.J. Singh[2,3]

*Authors contributed equally.

Correspondence to Dr. Maamed Mademilov; mademilov@gmail.com

[1] National Centre of Cardiology and Internal Medicine (NCCIM) named after academician M. Mirrakhimov - Bishkek (Kyrgyzstan)

[2] Department of Respiratory Sciences, University of Leicester (United Kingdom)

[3] Centre for Exercise and Rehabilitation Science, NIHR Leicester Biomedical Research Centre – Respiratory, University Hospitals of Leicester NHS Trust - Leicester (United Kingdom)

[4] Faculty of Health, University of Plymouth (United Kingdom)

[5] School of Sport, Exercise and Health Sciences, Loughborough University (United Kingdom)

Abstract

Objective

After experiencing tuberculosis (TB), many people develop post-tuberculosis lung disease (PTBLD). Pulmonary rehabilitation (PR) centrally comprising of education and exercise is recommended internationally for people living with chronic respiratory diseases (CRDs). However, no such service exists in Kyrgyzstan. This study investigated the opinions of healthcare professionals (HCPs) who would be expected to be potential future referrers to PR and adults living with PTBLD about what a PR program could look like in Kyrgyzstan.

Design

A qualitative study using interviews and focus groups. Grounded theory and thematic analysis were used for data collection and analysis.

Participants

63 participants; 15 referrers (12 male, 3 female; 12 pulmonolgists, 3 TB specialists) and 48 adults (26 male, 22 female) living with PTBLD.

For peer review only - http://bmjopen.bmj.com/site/about/guidelines.xhtml

Setting

Participants were recruited from hospital settings in Bishkek and Chuy Region, Kygryzstan.

Methods

Fifteen semi-structured interviews were conducted with referrers and 9 focus group discussions were conducted with adults living with PTBLD.

Results

Five key themes were developed; (i) living with PTBLD; (ii) attitude to PR, which emphasized the perceived importance and potential benefits of implemention; (iii) barriers/facilitators to PR, which included time and cost, and the importance of appropriate communication in enabling participation; (iv) interventional components of PR, which described culturally and demographically appropriate physical activities including rhythmic movements, dance, and volleyball; and (v) psychosocial support, which demonstrated the importance of psychological support for patients coping with the effects of stigma.

Conclusions

Potential referrers and adults living with PTBLD expressed their support for the implementation of PR. The culture-specific and population-specific issues highlighted in this work demonstrate the need to address stigma and provide certain types of exercise training/education modules for this specific clinical population. In other respects the currently known attitudes/barriers to PR, identified in Western research, appear to apply. The principles of culturally adapting PR may be helpful for those looking to establish similar clinical services in other Low-and Middle-Income Countries (LMIC) and in Central Asia in particular.

Data availability statement

Data relevant to the study has been included. Further data is available upon reasonable request.

For peer review only - http://bmjopen.bmj.com/site/about/guidelines.xhtml

Strengths and limitations of this study

- The opinions of patients with PTBLD and HCPs (TB specialists and pulmonologists) were deliberately taken into account to develop a pulmonary rehabilitation program.
- Participants were diverse in terms of demographic characteristics, severity of the disease, experience and social status.
- The study was conducted in one geographical region in Kyrgyzstan, therefore the implications reported need to be tested to show applicability to other cultural contexts.
- The study focused on PR for PTBLD rather than the broader spectrum of lung diseases.

Introduction

Worldwide, 10 million people suffer with tuberculosis (TB) and 1.5 million people die from TB annually. The burden of TB on individuals and society falls predominantly in low- and middle-income countries (LMIC)[1] where weak economic conditions aggravate the situation of people living with the disease, leading to disability and early mortality[2,3]. Kyrgyzstan is a LMIC in Central Asia with a high poverty level (20.1%)[4,5]. According to the World Health Organisation (WHO), the relative incidence rate of TB in Kyrgyzstan in 2019 was 144 per 100,000, the highest rate in the WHO European Region[6], as well as having one of the highest respiratory mortality rates[7].

Many adults who have experienced TB go on to develop post-TB lung disease (PTBLD). While chronic respiratory diseases (CRDs) are common in the Kyrgyzstan, PTBLD is the most challenging lung condition to address, because of public fears about the risk of infection to others, contagion which in turn leads to stigmatization, as evidenced by the issuing of a certificate of full recovery for these patients. People with PTBLD experience decreased exercise tolerance and increased cough, shortness of breath and general weakness. This is often accompanied by feelings of social isolation and depression[8,9]. There is evidence of persistent stigmatization of people living with TB even after recovery[10,11]; compounding problems such as social exclusion, unemployment, unstable housing and limited access to

For peer review only - http://bmjopen.bmj.com/site/about/guidelines.xhtml

medical care[10,12,13]. Depression, anxiety and somatic symptom disorders have a particularly negative impact on the standard of living of people in LMIC such as Kyrgyzstan[3,14,15].

Kyrgyzstan declared its independence from the Union of Soviet Socialist Republics (USSR) when it collapsed in 1991. The Soviet model of health care, which was centralized and provided universal free access to medical care, was designed for the prevention and early warning of diseases. During the Soviet era, people living with PTBLD across the USSR attended PR, where services ranged from three weeks to three months and included breathing exercises, prescribed walking, massage and various ball games. In addition, there were other alternative treatment institutions such as sanatoriums, salt mines and alpine resorts.

After the collapse of the Soviet Union, health care systems in independent states have experienced an economic crisis, an outflow of specialists, a decrease in the quality of medical care, a shortage of drugs, and an increase in the number of diseases such as TB and Human Immunodeficiency Virus (HIV). While the rehabilitation centers opened during the Soviet era continued to operate after the independence of Kyrgyzstan, due to a lack of funding, the quality and usefulness of the services provided has reduced[16]. Rehabilitation is rarely available to patients. Consequently, the burden of TB and PTBLD has continued to grow since Soviet times[17]. At present, the treatment process for these individuals is exclusively pharmacological (lasting between 6 months and 2 years) and, with no post-treatment care pathway, they are often left in a state of limbo whereby they are no longer infectious but are living with chronic symptoms and associated stigma. It is important to note that PR is not implemented anywhere in Central Asia.

The importance of pulmonary rehabilitation (PR) is well evidenced for people living with CRDs, including for adults living with PTBLD[10,18,19]. PR involves initial assessment followed by individualized therapy (exercise training and education) to improve physical and psychological well-being, and develop a long-term commitment to healthy behavior[20,21]. PR improves shortness of breath, exercise tolerance, and quality of life and reduces the number and duration of hospitalizations linked to exacerbations[18,22,23].

However, the introduction of PR in LMIC needs to be sensitive to both the local populations receiving care and the local context. Access to PR is typically through a referral process

For peer review only - http://bmjopen.bmj.com/site/about/guidelines.xhtml

whereby healthcare professionals (HCPs) introduce PR to patients. In many LMIC, including Kyrgyzstan, Pulmonologists and TB Specialists work directly with adults living with PTBLD and will likely be involved in referring patients for PR. Consequently, this study combines the views of potential PR referrers and adults living with PTBLD in Kyrgyzstan to identify both the potential acceptability and most appropriate design for PR.

Aims

To explore the views and experiences of potential referrers and adults living with PTBLD in Kyrgyzstan to inform the development of a culturally-appropriate PR service.

Methods

Patient and Public Involvement

As respiratory clinicians, we drew on our experiences of talking to people living with PTBLD when designing the study, in particular the interview schedule and logistics. Interviews and focus groups were organized based on work schedules and family commitments. This work was conducted to provide more in-depth insight into the views of patients to improve the quality of care they receive, such as the provision of appropriate PTBLD-specific PR. Patients were not directly involved in formulating the interview schedules or research question in the traditional sense of patient and public involvement. It was through informal conversations with patients and clinical consultations with patients that the importance of directly involving patients in the design of their PR arose; not least because of the stigma and alienation experienced as a result of their PTBLD. Patients who were involved in the research and resulting PR trial will be invited to support future dissemination activities. By the nature of our qualitative research, the findings of the present study are being used to develop a patient-informed PR programme in accordance with the principles of PPI. The findings of this study will be disseminated to participants and the wider public through awareness-raising activities in TB hospitals to help boost the profile of PR and inform the wider public about PTBLD-specific rehabilitation. The findings will also be disseminated through national and international conferences. The results of this study are expected to inform the Kyrgyz healthcare system by introducing a culturally-appropriate rehabilitation service informed by patients themselves.

For peer review only - http://bmjopen.bmj.com/site/about/guidelines.xhtml

For peer review only

Participants and data collection

In line with the research protocol, HCPs in regular contact with adults living with PTBLD were invited to participate and were recruited from 5 sites in the Chuy region that offer PTBLD treatment (National Hospital, National TB Hospital, City TB Hospital, Chuy region TB Hospital & Alamedin District TB Hospital). A total of 17 HCPs were invited to take part. Two HCPs declined to participate due to lack of time, leaving 15 individuals who participated in semi-structured interviews (12 females, 3 males; 3 Pulmonologists and 12 TB Specialists).

Participants for focus group (FG) discussions were invited from 8 sites (National Hospital, National TB Hospital, National Centre of Cardiology and Internal Medicine (NCCIM), City TB Hospital, Chuy region TB Hospital, Sokuluk District TB Hospital, Alamedin District TB Hospital & Kant District TB Hospital). All participants had previously received and finished basic treatment fo PTBLD. In total, 9 FGs were organized. 80 people were invited to participate, of whom 48 took part (26 men and 22 women) in groups of between 4 and 8. Participants came from a range of socio-economic backgrounds (including some homeless participants) and geographical locations (urban and rural).

The sampling strategy was a combination of targeted and convenience sampling. We targeted only medical specialists whose clinical practice directly involved dealing with PTBLD patients. All participants provided written informed consent. The interviews with referrers were conducted face-to-face at the participants' workplace in various health care facilities, both in urban (national center, city center) and rural settings between October 2019 and March 2020. The FGs were conducted on the premises of the department of respiratory medicine of the NCCIM between October 2019 and January 2020. During the FGs, participants were free to interact with each other, offer peer-to-peer support, ask and answer questions, and this method enabled patients to provide extended discussions of topics that were of particular interest to them, such as the problems around stigma. The interviewer (MM) was a Pulmonologist in Bishkek, Kyrgyzstan with previous experience in qualitative research. Interviewer MM acted as facilitator during focus groups. MM did not have any relationship with participants prior to study commencement and equally, participants did not know MM prior to the interviews.

For peer review only - http://bmjopen.bmj.com/site/about/guidelines.xhtml

The interview guides were structured using a narrative approach with additional prompts. Different interview guides were used for interviews and focus groups (See interview guides in supplemental appendix 1, 2). To improve and adapt questions and prompts, notes were taken and discussions of transcribed data were regularly held with the research team. The interviews and FGs were conducted in Russian and Kyrgyz languages according to participant preference. The researchers used audio recordings to collect the data. The average interview duration was 30 minutes, and for FGs 50 minutes. There were no follow-up interviews or FGs.

The the interview recordings were transcribed verbatim and then translated into English to enable the cross national team to fully participate in the analysis. The translation was carried out by a Kyrgyz team who were able to understand and interpret all expressions, and conveyed such details as laughter, expressions, etc. in the transcripts. Specialist and local terms were converted into an accessible form for understanding in English. Each interview and FG transcript was assigned a sequence number. Transcripts were anonymized and were not returned to participants for verification.

Data Analysis

The theoretical framework used was grounded theory and the data were analyzed using thematic analysis[24]. The flexibility and explorative character of thematic analysis makes it particularly appropriate for comparing and contrasting qualitative data drawn from different sample groups, and where researchers have variable levels of understanding of the life-worlds of different participant groups.

Each step of the analysis was carried out independently by the Kyrgyz and UK teams, followed by joint discussions, and agreement on the key themes. The first step involved reading and re-reading the transcripts several times (in their original or translated form), and writing a brief summary of the data. Step two involved manually creating codes (software packages were not used). There were seven coders in total; three in Kyrgyzstan, and four in the UK. The combination of similar phrases and sentences were highlighted in the same color, placed under the same code and a description of its contents was developed. A total of 21 initial codes were created. The third step involved refining the themes by grouping together similar codes. The fourth step involved collating all of the

For peer review only - http://bmjopen.bmj.com/site/about/guidelines.xhtml

relevant quotes and clarifying how each data extract related to the different themes. The fifth step involved developing a detailed description of themes and subthemes together with the use of relevant quotes.

Summary findings and interpretations encapsulating the perspectives of both referrers and adults living with PTBLD are presented. For the purposes of this article, data have been organized into five overarching themes, impacting a range of different PR-related issues. Quotes were selected from frequently encountered codes during the interviews and FGs and chosen as the best representation of the analytic point. Recruitment targets were determined a priori according to the norms of the respective methods. Partly due to the delays imposed by transcription, translation and cross-national analysis, but also due to the relative ease of access, we recruited up to these targets. Data saturation was achieved, but known only retrospectively as the data analysis progressed.

Results

Referrers and respondents living with PTBLD were positive about the potential introduction of PR. They identified a range of barriers/facilitators that would need to be considered in the design and implementation of any such program. A range of potential PR components were identified, as well as the need to tailor PR to individual physical conditions, provide effective communication and education, and be sensitive to the patients' psychosocial state. It was also noted the importance of including psychological support in the PR, which will be specific to patients with PTBLD who have stigma and psychological problems. Respondents living with PTBLD shared their illness experiences, such as symptom burden, challenges faced, and patterns of exercise and physical activity, and provided a vision of how a PR program should best be designed. Themes obtained in the thematic analysis are presented in the diagram (See diagram in supplemental appendix 3).

Living with PTBLD

Respondents living with PTBLD identified a range of expected symptoms including, "weakness and shortness of breath" (#FG2). However, the severity of symptoms varies for people living with PTBLD and affects their daily life in different ways. For some, symptoms do not present any difficulties for day-to-day living; "At the moment I am walking freely, doing household chores" (#FG5). But how strongly the respiratory symptoms of PTBLD

For peer review only - http://bmjopen.bmj.com/site/about/guidelines.xhtml

affect quality of life was noted by another respondent: "…with any physical movement I have shortness of breath… shortness of breath is strong" (#FG9).

Many respondents living with PTBLD worry about contracting TB again. They strictly monitor their symptoms and when their condition worsens or new symptoms emerge, they turn to medical staff for advice:

> "…I do everything that the doctor said, every year I go to the clinic to [the] TB specialist, cough, sneeze, get a yearly consultation, every year I go to X-rays" (#FG1)

In addition to recognizing the efficacy of traditional treatments, some respondents resort to alternative treatments; "…I drink rose hips, sometimes with lemon, I eat a spoonful of honey all the time, I try to drink milk, yogurt" (#FG1). Many respondents choose spa treatment: "… I went to the salt room, in the sanatorium, Kyrgyz seaside" (#FG1). Others noted that movement and physical exercise in general improved their well-being:

> "I…try to walk, sometimes I walk from morning to evening. I believe that walking, in general, physical activity is a huge benefit" (#FG5)

Particularly in rural communities, where agricultural work is common, day-to-day living is relatively active. Others noted how activity was embedded into their lifestyle - "I walk a lot, do not use the washing machine, cook and wash" (#FG5) – or did certain activities at home: "…I do breathing exercises so as not to cough. In these moments, gymnastics helps me" (#FG2).

Respondents have become accustomed to living with respiratory symptoms; "I wanted to live and breathe deeply, we have already forgotten what it is, but we want it so much" (#FG1). They are frequently proactive, seeking to improve their condition; "…we make different attempts, we want to get better. We need life" (#FG2). Consequently there is a significant demand for the implementation of a service like PR, which can help improve health-related quality of living.

Attitude to PR

All referrers noted the importance of implementing PR and recognized that the current absence of services meant that, post-treatment, people living with PTBLD were left without effective guidance:

For peer review only - http://bmjopen.bmj.com/site/about/guidelines.xhtml

> “Rehabilitation is the process necessary after completion of treatment for complete recovery” (#R12)

This sense of importance was confirmed by the FG data, whereby participants noted the potential of PR for improving their quality of life, increasing exercise tolerance and reducing the severity of symptoms: “If I constantly move, I feel good, nothing hurts” (#FG8). However, some have a limited awareness about which types of activities are suitable - “…you can or cannot play sports…, I don’t know…" (#FG4) - or how to participate in these activities safely; "…we don't know what movements to do and how to do them correctly" (#FG2).

Consequently, FG respondents noted the importance of receiving trustworthy, expert advice about PR and qualified assistance from medical personnel; “health professionals need to be trained so that I can come and ask, and not receive the answers that I know" (#FG1). HCPs should also adapt PR to individual needs: "The main thing is that the coach is good, that he knows how to give the right load" (#FG5).

Referrers noted the value of helping people to improve physical function and adapt to social life. As one referrer noted, PR would:

> “[lead to] an increase in tolerance to physical exertion, it would be like an addition to the main treatment… then it would reduce the number of complications, the number of exacerbations in general... so that the diseases do not become more complicated, do not worsen, as it seems to me that PR has a huge role [to play].” (#R9)

Respondents living with PTBLD generally exhibited a positive attitude towards PR with many expressing an interest in participating: "I will gladly come" (#FG1). Another respondent stated: “Yes, absolutely necessary" (#FG3). The FGs also revealed that some people might not want to take part in PR; for example, people who believe they are relatively healthy or have mild symptoms may be uninterested and see no benefit in participating.

Barriers/Facilitators to PR

HCPs identified relatively few barriers to referring patients to PR. Only one fundamental issue was identified as precluding PR participation; namely the presence of concomitant diseases (#R11) or unstable conditions (#R9).

For peer review only - http://bmjopen.bmj.com/site/about/guidelines.xhtml

Referrers did however note that some personality types – "phlegmatic people" (#R8), those whose behavior was erratic, "forgetful, shy" (#R11) - were not well suited to PR and that this would affect uptake. The importance of personality type was also noted by FG respondents:

> "Among the patients there are aggressive patients who run into others for no reason, and there are calm ones on the contrary, who prefer more privacy" (#FG7)

A further consideration was the social situation of some adults. It was noted that many migrate (primarily for economic reasons) after finishing treatment. It was also noted that, "among the patients with TB, we have former convicts and drug addicts" (#R10), "alcoholics, homeless people" (#R4) and so PR opportunities would not be taken up by everyone.

More generally, cost and time were identified as major considerations. A number of referrers argued that PR must be free to encourage attendance and meet the expectations of those living with PTBLD (#R5, 11, 13). FG respondents felt the same (#FG1) and emphasized the potential financial burden of implementing PR: "I wish there was a discount. Many patients do not work, there is little money…"(#FG2).

Personality types and the social situation were identified as interdependent factors. One referrer noted that attitudes from those with PTBLD were affected by the experience of having TB; "…because our patients have been treated for a long time, they are asocial for a very long time" (#R1). Others felt that the prolonged treatment period often experienced would make people reluctant to volunteer for further engagement. Some referrers noted that adherence could be affected by slow positive effects on health, which can lead to a loss of interest:

> "…they don't feel [the effects] right away… gradually if this is repeated regularly, you will feel a positive effect" (#R2)

The two primary solutions proposed for these issues were educating the patient with a "conversation about the need for post-treatment rehabilitation" (#R1), and attending to the "dynamics" and "subjective feelings" (#R2) generated in the PR environment that will help strengthen adherence to PR.

Referrers talked about the need for effective doctor-patient communication for the success of PR. In the context of referring a patient to PR, interviewees noted that the authority and

For peer review only - http://bmjopen.bmj.com/site/about/guidelines.xhtml

recommendations of the attending physician will have a very important impact on a person's decision to participate in PR:

> "If you explain normally to a person, any person will do what the doctor recommends, because in our society, as a doctor, as long as we have authority, patients believe us." (#R3)

Most respondents noted that they would follow recommendations to improve their health:

> "You need to do whatever your doctors tell you, take the right medication, eat right" (#FG3).

However, positive clinician-patient relations cannot be taken for granted as some of the FG respondents recalled unfavourable experiences with medical staff in the past. Being open and friendly was seen as an important source of motivation for those living with PTBLD;

> "…if you communicate openly with them, they are also open patients, and they will be happy to hear from you… happy to know any information." (#R2)

However, this alone would not be sufficient to mitigate experiences of stigma, which created a closed-minded attitude amongst those affected. Referrers explained how this could affect interactions with other HCPs:

> "…because even if we write a certificate for them, … [that] they are not contagious to others, sometimes in the consultation department or even in the hospital they look at patients differently and say 'you are ill with TB'" (#R12)

Interventional components of PR

While all referrers and FG respondents noted the importance of implementing physical exercises, a broad range of specific exercises were described. Referrers noted that walking was "the simplest and cheapest method" (#R2), while swimming and Nordic walking were commended for activating a wide range of muscles (#R9, #R15). Other activities included rhythmic movements (dancing, wushu [Chinese Kungfu]), ball sports (volleyball, basketball) and recreational activities (running, cycling, gymnastics). Like many countries, Kyrgyz culture is heavily influenced by dance, and the national dance "Kara Jorgo" ("Black Stallion") was cited as an activity that may be suitable to include in PR (#R12). Referrers argued for

For peer review only - http://bmjopen.bmj.com/site/about/guidelines.xhtml

“…almost all the exercises that involve the respiratory muscles, [and] the muscles of the shoulder girdle ... especially the muscles of the arms and shoulders” (#R9). They further noted the “…need to try everything…” (#R5) because different people like different forms of exercise.

Referrers and FG respondents also supported singing as part of the rehabilitation, though there were several opposing opinions. For instance, FG participants argued that “singing songs can be added to rehabilitation… it's good for the lungs” (#FG5), while referrers noted that attention should be paid to contraindications and severe concomitant diseases, which can lead to serious consequences such as internal bleeding:

> “If they have changes in the lungs, if they have caverns, then… singing can somehow affect the deterioration of their condition” (#R1)

But of greater concern to the referrers and FG respondents was that the intensity of activity should be suitable to the individual. Exercises needed to be done, “…not at such a pace, but … sparing, light” (#R4). As one FG respondent remarked, “…don't overwork" (#FG4). Referrers rejected “…the dances where they jump intensively there, run there,” (#R9) and recommended activities such as:

> “…rhythmic dances, then slow ... they can still use the respiratory muscles in their work and [this] may, it seems to me, be useful for recovery” (#R9)

Additionally, FG respondents emphasized the importance of developing the correct schedule and suggested having both morning and evening classes because, “sometimes they [people] are not allowed to leave work" (#FG1). Others noted that the time pressures experienced by those who work or have domestic or childcare duties meant that it would be good if “some exercises can be done at home” (#R11).

Finally, referrers stated that where education was offered to those with PTBLD, verbal delivery was preferable to reading or text:

> “…video lectures should be organized in all clinical specialized pulmonary units. They should be concise, lasting 10-15 minutes” (#R6)

For peer review only - http://bmjopen.bmj.com/site/about/guidelines.xhtml

Despite various challenges, the strength of desire to overcome the consequences of PTBLD was evident in the respondents' willingness to spend their limited time and money to participate in PR (#FG1). The benefits of PR were highlighted: "the time spent on recovery is not in vain. To do this, you can postpone other activities" (#FG5).

Psychosocial support

All TB specialists noted the need to address psychological problems (e.g. depression, becoming withdrawn or aggressive), personal and relational issues (e.g. with family) and to help people living with PTBLD adapt to social life. According to referrers, people may experience stigmatization from a variety of sources. Some people "leave the family" (#R13) due to the stigma of illness, while others are afraid that they will meet people they know at the hospital; "…[patients] tell us where and in which hospitals their relatives, friends work and do not want to visit [these places]' (#R15). Another noted that, "stigma discrimination is very common among our patients, and I think that psychosocial support can be connected to PR" (#R2).

This was supported by the FG data, where people shared their experiences of PTBLD-related stigma. Several participants said they experienced difficulties finding a job; "I tried so many times to get a job, they didn't take me" (#FG7). Some people struggle to recover and after treatment and discharge, they are subjected to stereotyping, affecting their relationships with their loved ones. For example,

> "My neighbor...told her (daughter-in-law) and the children not to come to me, that I was contagious, and to other neighbors that I needed to be evicted." (#FG3)

Indicatively, it was suggested PR should be done "anonymously so that no one knows" (#FG1).

Consequently, both referrers and FG participants placed significant emphasis on the role of psychological support within PR interventions. It was argued that some psychological support would be provided through the social nature of some PR activities. Where rehabilitation takes place in a group, all adults with PTBLD "will be equal … to each other, they will talk, they will communicate" (#R3). Conducting group PR discussions could provide mutual support because, "…until a person gets sick, they do not understand others, and

For peer review only - http://bmjopen.bmj.com/site/about/guidelines.xhtml

there everyone understands each other, they talk, they share secrets” (#R11). There was also a call for more formal, professional psychological support; "we need psychological therapy" (#FG7).

Thus, it was not only argued that PR needed to incorporate psychological support but that it offers a good opportunity to provide the kind of support that was more generally needed by people living with PTBLD. As one FG respondent noted, "The main thing is psychological support. Sport is sport, but warm words cannot be replaced by anything" (#FG5).

Discussion

The need to develop appropriate PR services for people living with PTBLD, and who reside in LMIC, is an important issue that has scarcely been studied. Kyrgyzstan is leading the development of PR among many other post-Soviet countries and in Central Asia, where there is currently an absence of rehabilitation services. Consequently, about how to successfully implement PR may have significant implications for other LMIC or low-resource contexts. This qualitative study synthesized the views of adults living with PTBLD, and the TB Specialists and Pulmonologists who would initiate referral, to explore how PR could be tailored to maximize uptake in the context of Kyrgyzstan. The results indicate both a general need and willingness to embrace PR, as well as key barriers (e.g., cost, time) and facilitators (e.g. staffing, scheduling, and delivery mode) to uptake, and specific guidance on the components of intervention, including the role of psychosocial support. PR was viewed as playing an important role in improving both people’s physical and mental health.

The integration of PR into the treatment pathway should be supported by strong long-term relationships between patients and clinicians, primarily a TB specialist or Pulmonologist. The data suggest that a general inclination to respect the authority of medical professionals, and a widespread compliance with treatment recommendations, will be enhanced by an open and honest attitude towards patients. Good communication by HCPs is needed to build rapport and trust with a cohort who may have experienced discrimination. There is a need to include PR in doctor-patient conversations early in the TB treatment process so that those who go on to develop PTBLD are aware that PR is a necessary and valuable part of their overall recovery and reintegration into society. Because of the contrasting medical guidance at the treatment and rehabilitation phases for TB (i.e. initially rest and latterly

For peer review only - http://bmjopen.bmj.com/site/about/guidelines.xhtml

activity), clinicians should be transparent and consistent in promoting awareness of the benefits of PR early on during treatment. This will likely boost the uptake to PR.

In particular, continued patient engagement will be influenced by social factors. Because social attitudes prevent some patients from participating in hospital-based PR services, it is vital to design a recruitment pathway and appointment logistics that are sensitive to issues relating to stigmatization. For example, it may be suitable to conduct PR classes at quieter times of the day, to help patients arrange transport or to offer overnight accommodation for those travelling far from rural regions. It would be desirable to provide advice about exercise that can be done at home and particularly about what level of exertion is both beneficial and safe. Many people living with PTBLD experience financial difficulties[25], and either struggle to find employment or secure only relatively low-paid jobs. Therefore, when organizing PR for such a population, discounted or low-cost provision is essential. It is significant that participants preferred modes of exercise training that do not require expensive equipment and could be undertaken in home or community settings, such as walking, dance and balls sports as this facilitates the implementation of PR in LMIC.

Educational components of PR should be tailored to the needs of the particular audience. In parts of the world where PR is part of standard care, education has traditionally been delivered in a lecture style but the data suggest that information provided verbally, with supporting images, and in ‘bite size’ amounts, would be more suitable for the PTBLD population in Kyrgyzstan. This fits with an ongoing transformation of PR education delivery in other parts of the world, such as in the UK, to move away from didactic deliveries, with the educational and cultural backgrounds of PR attendees considered[26].

Many previous studies have focused on stigma related to the infectious nature of TB and the lack of awareness within the general population that these individuals are no longer contagious after completing their drug treatment[11]. Studies conducted in LMIC such as Vietnam[27] and India[11], reported that TB is often deemed a ‘death penalty’ and perceived as a ‘dirty disease’. Studies in LMIC have shown that the effects of stigma can both influence a patient’s treatment and care-seeking behavior[28,29] and undermine patients’ trust in HCPs[30]. A previous Kyrgyzstan study found that TB patients frequently experience discrimination from HCPs who sought to avoid interaction[15].

For peer review only - http://bmjopen.bmj.com/site/about/guidelines.xhtml

A major lesson from this study is that individuals with PTBLD may continue to experience discrimination, including from HCPs who deny them advice and make it difficult for them to visit healthcare facilities. PR offers an opportunity for these individuals to receive care from HCPs free from prejudice. Incorporating educational sessions will help them understand their disease and the social attitudes they experience. In this regard, both sets of respondents emphasized the importance of incorporating psychosocial support as part of PR to help people cope with the effects of stigma. Incorporating group sessions in a safe, non-judgmental environment offers peer-peer support and will enable patients to share their experiences openly.

Another way to promote PR attendance and adherence is to embed culturally and demographically-appropriate activities. The core PR components of aerobic exercise typically include walking/cycling, strength training and education[18]. The results of this study suggest that the generic principles of PR can be adapted to the local context or population. For example, respondents spoke about the popularity of ball sports such as volleyball, reminiscent of the rehabilitation offered in the Soviet era. Conventional exercises like squats can be 'gamified' into a volleyball dig action to increase enjoyment and motivation. Adaptations are likely to be most successful where they also help patients' understanding of how these exercises can translate into benefits in daily life e.g. taking part in social activities or preparing for employment.

Respondents supported the incorporation of rhythmic movements such as dance performed at an appropriate intensity. A national dance such as Kara Jorgo, can both be scaled to each person's physical condition and offer a culturally relevant and enjoyable form of exercise to strengthen muscles involved in the mechanics of breathing. Dance has been shown to improve health, including physical performance and mood[31,32]. Taking part in socially conventional exercises will challenge stigma and show people living with PTBLD how PR is also relevant to everyday living.

In addition to dancing, singing is also an important part of the Kyrgyz culture. Unlike dancing, singing had a mixed reception from participants some of whom were enthusiastic and some of whom said it would not be popular[33]. Although singing therapy has been tested previously, with evidence suggesting that it can improve wellbeing for adults with COPD[34,35],

For peer review only - http://bmjopen.bmj.com/site/about/guidelines.xhtml

evidence relating to the effects of singing for PTBLD populations is lacking. Consequently, further research is required before implementing singing as part of PR in the contexts of PTBLD and Central Asia.

Limitations

The study was conducted in one geographical region in Kyrgyzstan, therefore the applicability of the implications reported require empirical verification. The absence of holistic treatment for this group of patients and the problems of care do not provide a complete picture to understand their demographic characteristics and severity of the disease, and thus it is unclear if the study results can be generalized to other people with PTBLD in Kyrgyzstan. The study focused on PR for PTBLD rather than the broader spectrum of lung diseases, but it is important to note that there is no evidence to suggest that the implementation of PR for PTBLD is likely to be easier in relation to this group, due to the stigma associated with the condition.

Conclusion

PR is embedded in routine clinical care in Western countires with unequivocal evidence of its benefits. In this qualitative exploration, potential PR referrers (TB Specialists and Pulmonologists), as well as adults living with PTBLD, were overwhelmingly positive towards the implementation of PR in Kyrgyzstan. The culture-specific and population-specific issues highlighted in this work demonstrate the need to address stigma and provide certain types of exercise training/education modules for this specific clinical population. In other respects the currently known attitudes/barriers to PR, identified in Western research, appear to apply. The act of culturally adapting PR may be beneficial for other settings looking to establish PR as a clinical service or evaluate PR in clinical trials in low resource settings. The results of this study can be applied to provide a basis for the implementation of PR programs and to evaluate the effectiveness of such programs for people with PTBLD. Future research should focus on the geographical generalizability of these findings, and the applicability for PR programs for other CRDs.

For peer review only - http://bmjopen.bmj.com/site/about/guidelines.xhtml

Footnotes

Ethics statements

Ethics approval

This work is part of NIHR Global Health Research Group on Respiratory Rehabilitation (Global RECHARGE; 17/63/20), approved by The National Center for Cardiology and Internal Medicine (NCCIM; ref: 17) and University of Leicester (ref: 22293) ethics committees. This study was registered prospectively (ISRCTN16501214).

Funding: This research was funded by the National Institute for Health Research (NIHR) (17/63/20) using UK aid from the UK Government to support global health research.

Acknowledgments

Competing Interest statement

All authors declare no competing interest.

Contributorship statement

M. Mademilov - conducting interviews (data collection), thematic analysis, data interpretation and writing an article

G. Mirzalieva - conducting interviews (data collection), supporting thematic analysis, data interpretation and writing an article

Z.K. Yusuf - supporting thematic analysis, data interpretation and writing an article

M.W. Orme - supporting thematic analysis, data interpretation and writing an article

C.L.A. Bourne - supporting thematic analysis and writing an article

A. Akylbekov - supporting thematic analysis and writing an article

A.V. Jones - assisting with interview scheduling and data interpretation

R.B. Miah - assisting with interview scheduling and data interpretation

R. Jones - supporting with data interpretation and data analysis

A. Barton - supporting thematic analysis and writing an article

D. Malcolm - supporting thematic analysis, data interpretation and writing an article

T. Sooronbaev - development of the concept and design of the study and writing an article

S.J. Singh - development of the concept and design of the study and writing an article

For peer review only - http://bmjopen.bmj.com/site/about/guidelines.xhtml

For peer review only

Data availability statement

Data are available upon reasonable request

References

1. Tuberculosis. *World Health Organization*, World Health Organization, 2019, www.who.int/health-topics/tuberculosis#tab=tab_1.

2. Jaramillo, E. Pulmonary tuberculosis and health-seeking behaviour: how to get a delayed diagnosis in Cali, Colombia. *Tropical medicine & international health: Trop. Med. Int. Heal.* (1998); 3.2: 138-144.

3. Peltzer, K. *et al.* Prevalence of psychological distress and associated factors in tuberculosis patients in public primary care clinics in South Africa. *BMC psychiatry* (2012);12.1: 1-9.

4. About Us: UNDP in Kyrgyz Republic. *UNDP*, 2015, www.kg.undp.org/content/kyrgyzstan/en/home/about-us.html#:~:text=Kyrgyzstan is a landlocked%2C largely,income of %241%2C170 in 2015.

5. Living standard. *National Statistical Committee of the Kyrgyz Republic*, 2019. http://www.stat.kg/en/statistics/uroven-zhizni-naseleniya/.

6. Tuberculosis Surveillance and Monitoring in Europe, 2019. *European Centre for Disease Prevention and Control*, www.ecdc.europa.eu/en/publications-data/tuberculosis-surveillance-and-monitoring-europe-2019.

7. Gibson, J., *et al.* The Burden of Lung Disease. *ERS*, 2013, www.erswhitebook.org/chapters/the-burden-of-lung-disease/.

8. Muñoz-Torrico, M. *et al.*, Is there a rationale for pulmonary rehabilitation following successful chemotherapy for tuberculosis?, *J. Bras. Pneumol.* (2016); 42.5: 374–385, 2016.

9. Cheng-Ting, L. *et al.* Evaluation of health-related quality of life in patients with tuberculosis who completed treatment in Kiribati. *Journal of International Medical Research* (2017); 45. 2: 610-620.

10. Allwood, B.W *et al.*, Post-tuberculosis lung health: Perspectives from the First International Symposium. *Int. J. Tuberc. Lung Dis.* (2020); 24. 8: 820–828.

11. Rajeswari, R. *et al.* Perceptions of tuberculosis patients about their physical, mental and social well-being: a field report from south India. *Soc. Sci. Med.* (2005); 60. 8: 1845–1853.

12. Datiko, D.G., Jerene, D., and Suarez, P. Stigma matters in ending tuberculosis: Nationwide survey of stigma in Ethiopia. *BMC Public Health* (2020); 20. 1: 1–10.

13. Bauer, M. *et al.*, Health-related quality of life and tuberculosis: A longitudinal cohort study. *Health Qual. Life Outcomes* (2015); 13.1: 1–13.

For peer review only - http://bmjopen.bmj.com/site/about/guidelines.xhtml

14. Walker, I. F., *et al.* Implementation of a psychosocial support package for people receiving treatment for multidrug-resistant tuberculosis in Nepal: A feasibility and acceptability study. *PloS one* (2018); 13.7: e0201163.

15. Burtscher, D. et al. 'My favourite day is sunday': Community perceptions of (drug-resistant) tuberculosis and ambulatory tuberculosis care in Kara Suu District, Osh Province, Kyrgyzstan. *PLoS One*, (2016); 11. 3: 1–16.

16. Rechel, B. Health policy reform in the countries of the former Soviet Union. *The Palgrave International Handbook of Healthcare Policy and Governance*. Palgrave Macmillan, 2015.

17. Arinaminpathy, N. and Dye, C. Health in financial crises: Economic recession and tuberculosis in Central and Eastern Europe. *J. R. Soc. Interface* (2010): 7. 52: 1559–1569, 2010.

18. McCarthy, B. *et al*. Pulmonary rehabilitation for chronic obstructive pulmonary disease. *Cochrane database Syst. Rev.* (2015); 2: CD003793.

19. Singh, S.K., *et al.* Pulmonary Rehabilitation in Patients with Chronic Lung Impairment from Pulmonary Tuberculosis. *Cureus* (2018); 10. 11.

20. Jones, R. *et al*. Does pulmonary rehabilitation alter patients' experiences of living with chronic respiratory disease? A qualitative study. *Int. J. COPD* (2018); 13: 2375–2385.

21. Corhay, J.L., *et al.* Pulmonary rehabilitation and copd: Providing patients a good environment for optimizing therapy. *Int. J. COPD* (2013); 9: 27–39.

22. Griffiths, T.L., *et al.*, Results at 1 year of outpatient multidisciplinary pulmonary rehabilitation: a randomised controlled trial. *Lancet* (2000); 355. 9201: 362–368.

23. Puhan, M.A. *et al*. Pulmonary rehabilitation following exacerbations of chronic obstructive pulmonary disease. *Cochrane Database Syst. Rev.* (2016); 12.

24. Braun, V., and Clarke, V. Using thematic analysis in psychology. *Qualitative research in psychology* (2006); 3. 2: 77-101.

25. Tanimura, T. *et al.* Financial burden for tuberculosis patients in low-and middle-income countries: a systematic review. *European Respiratory Journal* (2014); 43.6: 1763-1775.

26. Bolton, C.E., *et al.* British Thoracic Society guideline on pulmonary rehabilitation in adults: accredited by NICE. *Thorax* (2013); 68. 2: 1-30.

27. Hoa, N.P., *et al.* Knowledge of tuberculosis and associated health-seeking behaviour among rural Vietnamese adults with a cough for at least three weeks. *Scandinavian Journal of Public Health* 2003; 31. 62: 59-65.

28. Ngamvithayapong, J., Winkvist, A. and Diwan, V.. High AIDS awareness may cause tuberculosis patient delay: results from an HIV epidemic area, Thailand. *Aids* (2000); 14. 10: 1413-1419.

29. Weiss, M.G., Ramakrishna, J. and Somma, D. Health-related stigma: rethinking concepts and interventions. *Psychology, health & medicine* (2006); 11.3: 277-287.

30. Miller J A. Perception and beliefs of health care workers about clients with tuberculosis. Auckland: University of Auckland; 2007.

For peer review only - http://bmjopen.bmj.com/site/about/guidelines.xhtml

31. Koch, S.C., *et al.* Effects of dance movement therapy and dance on health-related psychological outcomes. A meta-analysis update. *Frontiers in psychology*, (2019); 10: 1806.

32. Williams, S. Dance group enriches lives of people living with breathlessness. *The Lancet Respiratory Medicine* (2020); 8. 8: 763-764.

33. McNamara RJ *et al.* Singing for adults with chronic obstructive pulmonary disease (COPD). *Cochrane Database Syst Rev.* (2017); 12.

34. Lord, V.M., *et al.* Singing classes for chronic obstructive pulmonary disease: a randomized controlled trial. *BMC Pulmonary Medicine* (2014); 14: 181-181.

35. Downes, C. *et al.* Singing for Breathing Uganda: Group singing for people with chronic lung disease in Kampala. *Journal of Applied Arts & Health* (2019); 10. 2: 219-228.

For peer review only

For peer review only - http://bmjopen.bmj.com/site/about/guidelines.xhtml

Work Package 1 Patient Focus Group Schedule

The impact has PTBLD has had on their life

- When were you first diagnosed? What was it like to hear that?
- How did you expect PTBLD was going to affect you? Is there anything you wish you had been told about PTBLD? If so, what? Why? How would this have helped you?
- Did you know anyone else who had PTBLD? How are they treated by other people?
- How do you think other people have reacted to you having PTBLD? Did you tell family/friend/work colleagues? Immediately or not?
- What has most surprised you about having PTBLD? What are the differences between what people generally think about PTBLD and the actual experience of having it?
- Consider the impact on your work (employment), your domestic tasks, and leisure choices. What has been the most significant? What? It what ways? Give examples.
- What changes, if any, have you had to make to your life because of PTBLD? Prompt: employment, domestic duties, leisure activities, personal relationships?
- How would you advise others living with PTBLD?
- How do you feel about the future?

Everyday activities:

- When, in your everyday life are you most active?
- Do you prefer individual or social activities when you are being active? Why?
- Are there any activities that you would like to do? Or any that you no longer do that you would like to be able to do again?
- Other participants have talked about activities such as ball sports (e.g., basketball, volleyball, football), wushu, dancing, singing and music – what do you think about doing these activities?

Interviewer: Provide a brief overview of PR: "PR consists of an exercise and education programme. Treatment is tailored to each person and it aims to help you control the symptoms of your condition, educate you about your condition, improve general health and level of fitness, improve your ability to carry out daily activities that are important to you and improve your confidence and quality of life."

- What are your initial thoughts?
- How would you hope PR might help you? What would you hope to achieve from taking part?
- What do we need to think about in making you more comfortable to attend PR?
- Would you be sacrificing anything to be part of this? Time? Money? Energy that will stop you doing other things? What are the negative sides of doing PR?
- What do you think should be included in PR? Why? (PROMPT: psychological support, education, information, types of exercises). **Note: ask them for specific examples**
- Are there any activities or hobbies that could be useful in PR? (PROMPT: ball sports such as basketball, volleyball, football; wushu; dancing; singing and music
- What do you think the challenges might be with these activities as part of PR?

For peer review only

Work Package 2 Referrer Interview Schedule

Aim 1) General opinions about pulmonary rehabilitation

- What does the term pulmonary rehabilitation mean to you?
- What is your opinion about pulmonary rehabilitation?

  [Probing question: How important do you think it is to provide pulmonary rehabilitation?]
- Do you have any experience in rehabilitation?

  If so: What did the rehabilitation consist of?

  How long was the average course of rehabilitation?

  Roughly what percentage of patients would take part in the rehabilitation?
- What would you think about being involved in referring patients to pulmonary rehabilitation?
- What do you think about using your clinical time for pulmonary rehabilitation?
- What do you think the problems will be to referring patients to pulmonary rehabilitation?
- How do you think these problems can be resolved?

  [Probing question: What will be your reasons for referring patients to pulmonary rehabilitation?]
- How would you describe pulmonary rehabilitation to your patients?
- What characteristics will you look for when deciding whether or not to refer a patient?

  [Probing question: Are there certain types of patients you wouldn't even discuss pulmonary rehabilitation with? Why?]
- How do you know if someone is willing or ready to take part in pulmonary rehabilitation?
- Who do you think should be involved in assessing which pulmonary rehabilitation activities would help a specific patient? And why?
- What staff should be involved in providing pulmonary rehabilitation?

For peer review only

Aim 2) Patients' opinion of pulmonary rehabilitation

- How do you think patients will respond to your referral for pulmonary rehabilitation? Why?
- How do you think patients will get on with pulmonary rehabilitation? Why?
- What can we do to help patients complete their course of pulmonary rehabilitation?

Aim 3) Changes needed for pulmonary rehabilitation to work well in Kyrgyzstan

- What physical activities/exercise are good for these patients? Why? How might we do these activities as part of pulmonary rehabilitation?
- What do you think should be included in pulmonary rehabilitation? Why?

  [PROMPT: Education, information, types of exercises (e.g., walking, ball sports, cycling, yoga), psychological assistance, music]

  [Probing question: Why? Why not other activities?]
- What problems are caused by the stigma associated with PTBLD?

  [Probing question: How might these problems impact participation in pulmonary rehabilitation? What can we do to help patients take part? How can we help them to have a positive experience?]

  [PROMPT: environment, social influence, emotional conditions, feeling comfortable]
- Should pulmonary rehabilitation address problems about stigma?

  [Probing question: How might pulmonary rehabilitation reduce stigma?]

For peer review only

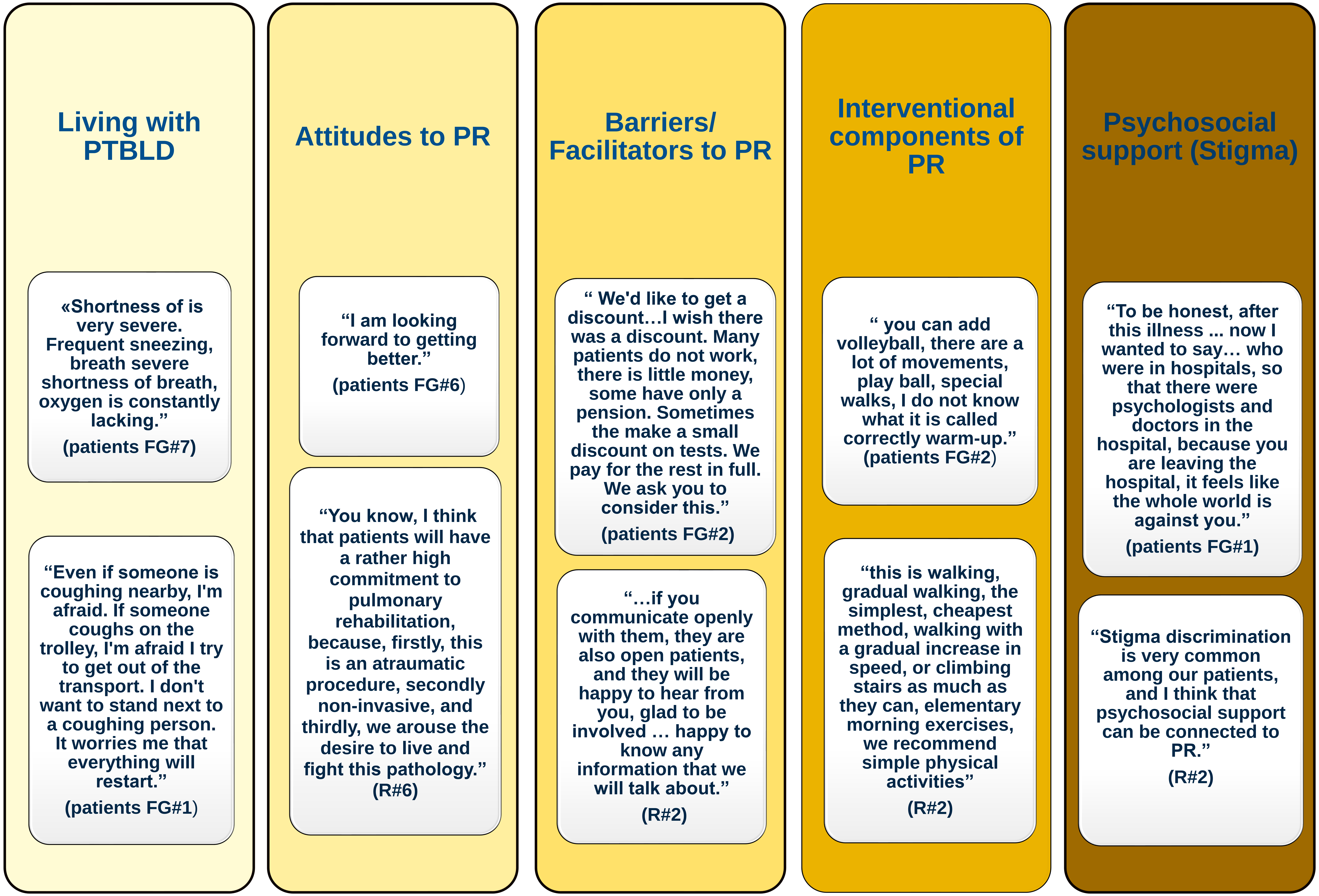
Living with PTBLD
«Shortness of is very severe. Frequent sneezing, breath severe shortness of breath, oxygen is constantly lacking.”
(patients FG#7)
“Even if someone is coughing nearby, I'm afraid. If someone coughs on the trolley, I'm afraid I try to get out of the transport. I don't want to stand next to a coughing person. It worries me that everything will restart.”
(patients FG#1)
Attitudes to PR
“I am looking forward to getting better.”
(patients FG#6)
“You know, I think that patients will have a rather high commitment to pulmonary rehabilitation, because, firstly, this is an atraumatic procedure, secondly non-invasive, and thirdly, we arouse the desire to live and fight this pathology.”
(R#6)
Barriers/ Facilitators to PR
“ We'd like to get a discount…I wish there was a discount. Many patients do not work, there is little money, some have only a pension. Sometimes the make a small discount on tests. We pay for the rest in full. We ask you to consider this.”
(patients FG#2)
“…if you communicate openly with them, they are also open patients, and they will be happy to hear from you, glad to be involved … happy to know any information that we will talk about.”
(R#2)
Interventional components of PR
“ you can add volleyball, there are a lot of movements, play ball, special walks, I do not know what it is called correctly warm-up.”
(patients FG#2)
“this is walking, gradual walking, the simplest, cheapest method, walking with a gradual increase in speed, or climbing stairs as much as they can, elementary morning exercises, we recommend simple physical activities”
(R#2)
Psychosocial support (Stigma)
“To be honest, after this illness ... now I wanted to say… who were in hospitals, so that there were psychologists and doctors in the hospital, because you are leaving the hospital, it feels like the whole world is against you.”
(patients FG#1)
“Stigma discrimination is very common among our patients, and I think that psychosocial support can be connected to PR.”
(R#2)

What should pulmonary rehabilitation look like for people living with post-tuberculosis lung disease in the Bishkek and Chui region of the Kyrgyz Republic?: A qualitative exploration

Standards for Reporting Qualitative Research (SRQR)*

http://www.equator-network.org/reporting-guidelines/srqr/

Title and abstract

	Page/line no(s).
**Title** - Concise description of the nature and topic of the study Identifying the study as qualitative or indicating the approach (e.g., ethnography, grounded theory) or data collection methods (e.g., interview, focus group) is recommended	1/ 1
**Abstract** - Summary of key elements of the study using the abstract format of the intended publication; typically includes background, purpose, methods, results, and conclusions	1/ 16

Introduction

**Problem formulation** - Description and significance of the problem/phenomenon studied; review of relevant theory and empirical work; problem statement	3/1
**Purpose or research question** - Purpose of the study and specific objectives or questions	4/23

Methods

**Qualitative approach and research paradigm** - Qualitative approach (e.g., ethnography, grounded theory, case study, phenomenology, narrative research) and guiding theory if appropriate; identifying the research paradigm (e.g., postpositivist, constructivist/ interpretivist) is also recommended; rationale**	7/2
**Researcher characteristics and reflexivity** - Researchers' characteristics that may influence the research, including personal attributes, qualifications/experience, relationship with participants, assumptions, and/or presuppositions; potential or actual interaction between researchers' characteristics and the research questions, approach, methods, results, and/or transferability	6/11
**Context** - Setting/site and salient contextual factors; rationale**	5/18
**Sampling strategy** - How and why research participants, documents, or events were selected; criteria for deciding when no further sampling was necessary (e.g., sampling saturation); rationale**	6/1
**Ethical issues pertaining to human subjects** - Documentation of approval by an appropriate ethics review board and participant consent, or explanation for lack thereof; other confidentiality and data security issues	18/17

For peer review only - http://bmjopen.bmj.com/site/about/guidelines.xhtml

For peer review only

**Data collection methods** - Types of data collected; details of data collection procedures including (as appropriate) start and stop dates of data collection and analysis, iterative process, triangulation of sources/methods, and modification of procedures in response to evolving study findings; rationale**	5/17
**Data collection instruments and technologies** - Description of instruments (e.g., interview guides, questionnaires) and devices (e.g., audio recorders) used for data collection; if/how the instrument(s) changed over the course of the study	6/15
**Units of study** - Number and relevant characteristics of participants, documents, or events included in the study; level of participation (could be reported in results)	5/23,28
**Data processing** - Methods for processing data prior to and during analysis, including transcription, data entry, data management and security, verification of data integrity, data coding, and anonymization/de-identification of excerpts	6/24<br>7/1
**Data analysis** - Process by which inferences, themes, etc., were identified and developed, including the researchers involved in data analysis; usually references a specific paradigm or approach; rationale**	7/2
**Techniques to enhance trustworthiness** - Techniques to enhance trustworthiness and credibility of data analysis (e.g., member checking, audit trail, triangulation); rationale**	7/7

Results/findings

**Synthesis and interpretation** - Main findings (e.g., interpretations, inferences, and themes); might include development of a theory or model, or integration with prior research or theory	7/28
**Links to empirical data** - Evidence (e.g., quotes, field notes, text excerpts, photographs) to substantiate analytic findings	8/21

Discussion

**Integration with prior work, implications, transferability, and contribution(s) to the field** - Short summary of main findings; explanation of how findings and conclusions connect to, support, elaborate on, or challenge conclusions of earlier scholarship; discussion of scope of application/generalizability; identification of unique contribution(s) to scholarship in a discipline or field	14/21
**Limitations** - Trustworthiness and limitations of findings	17/19

Other

**Conflicts of interest** - Potential sources of influence or perceived influence on study conduct and conclusions; how these were managed	18/25
**Funding** - Sources of funding and other support; role of funders in data collection, interpretation, and reporting	18/22

For peer review only - http://bmjopen.bmj.com/site/about/guidelines.xhtml

*The authors created the SRQR by searching the literature to identify guidelines, reporting standards, and critical appraisal criteria for qualitative research; reviewing the reference lists of retrieved sources; and contacting experts to gain feedback. The SRQR aims to improve the transparency of all aspects of qualitative research by providing clear standards for reporting qualitative research.

**The rationale should briefly discuss the justification for choosing that theory, approach, method, or technique rather than other options available, the assumptions and limitations implicit in those choices, and how those choices influence study conclusions and transferability. As appropriate, the rationale for several items might be discussed together.

Reference:

O'Brien BC, Harris IB, Beckman TJ, Reed DA, Cook DA. **Standards for reporting qualitative research: a synthesis of recommendations.** *Academic Medicine*, Vol. 89, No. 9 / Sept 2014 DOI: 10.1097/ACM.0000000000000388

For peer review only

For peer review only - http://bmjopen.bmj.com/site/about/guidelines.xhtml
